# Identification of the Key Parameters for Horizontal Transition Dipole Orientation in Fluorescent and TADF Organic Light‐Emitting Diodes

**DOI:** 10.1002/adma.202100677

**Published:** 2021-08-02

**Authors:** Francisco Tenopala‐Carmona, Oliver S. Lee, Ettore Crovini, Ana M. Neferu, Caroline Murawski, Yoann Olivier, Eli Zysman‐Colman, Malte C. Gather

**Affiliations:** ^1^ Organic Semiconductor Centre SUPA School of Physics and Astronomy University of St Andrews St Andrews KY16 9SS UK; ^2^ Humboldt Centre for Nano‐ and Biophotonics Department of Chemistry University of Cologne Greinstr. 4‐6 50939 Köln Germany; ^3^ Organic Semiconductor Centre EaStCHEM School of Chemistry University of St Andrews St Andrews KY16 9ST UK; ^4^ Unité de Chimie Physique Théorique et Structurale & Laboratoire de Physique du Solide Namur Institute of Structured Matter Université de Namur Rue de Bruxelles, 61 Namur 5000 Belgium; ^5^ Present address: Kurt‐Schwabe‐Institut für Mess‐ und Sensortechnik Meinsberg e.V. Kurt‐Schwabe‐Straße 4 Waldheim 04736 Germany

**Keywords:** meta‐analysis, molecular orientation, multiple linear regression, organic light‐emitting diodes, thermally activated delayed fluorescence

## Abstract

In organic light‐emitting diodes (OLEDs), horizontal orientation of the emissive transition dipole moment (TDM) can improve light outcoupling efficiency by up to 50% relative to random orientation. Therefore, there have been extensive efforts to identify drivers of horizontal orientation. The aspect ratio of the emitter molecule and the glass‐transition temperature (*T*_g_) of the films are currently regarded as particularly important. However, there remains a paucity of systematic studies that establish the extent to which these and other parameters control orientation in the wide range of emitter systems relevant for state‐of‐the‐art OLEDs. Here, recent work on molecular orientation of fluorescent and thermally activated delayed fluorescent emitters in vacuum‐processed OLEDs is reviewed. Additionally, to identify parameters linked to TDM orientation, a meta‐analysis of 203 published emitter systems is conducted and combined with density‐functional theory calculations. Molecular weight (MW) and linearity are identified as key parameters in neat systems. In host–guest systems with low‐MW emitters, orientation is mostly influenced by the host *T*_g_, whereas the length and MW of the emitter become more relevant for systems involving higher‐MW emitters. To close, a perspective of where the field must advance to establish a comprehensive model of molecular orientation is given.

## Graphical abstract

Horizontal emitter orientation can significantly increase the efficiency of organic light‐emitting diodes. Intense research over the past decade has provided a large amount of data on the factors that may drive horizontal orientation of organic emitters. This research is reviewed, and a meta‐analysis of the data is combined with quantum‐chemical calculations to identify key parameters that influence this orientation.

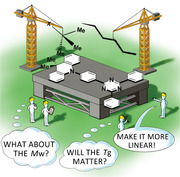

## 1 Introduction

Organic light‐emitting diodes (OLEDs) are electroluminescent solid‐state light sources in which the active light‐emitting materials consist of organic or organometallic molecules, dendrimers, or polymers containing conjugated π‐electron systems. The extended π‐conjugation of these materials renders them semiconducting, and modulation of the π‐conjugation allows tunable luminescence across the entire visible range of the electromagnetic spectrum. At the same time, many organic semiconductors show mechanical flexibility and an amorphous structure. These properties make them well suited for producing optoelectronic devices with an attractive combination of properties, such as mechanical flexibility, versatile form factors (including large area), and compatibility with a variety of substrates. In the more than three decades since the first OLED was reported,^[^ ^1^ ^]^ progress has been dramatic and these devices are now finding numerous commercial applications: a substantial fraction of modern smartphone displays are based on OLEDs, large‐area OLED TVs are rapidly gaining in popularity, and OLED‐based luminaires may well provide glare‐free illumination in offices and homes in the near future. While most of the current commercial devices do not yet exploit the possibility of making mechanically flexible devices, they strongly benefit from a lower production cost per area than, for example, epitaxial growth of conventional inorganic LEDs, as well as from the ability to directly deposit OLEDs on the large thin‐film transistor backplanes used in the display industry. In the future, the ability to produce thin, large area, and flexible light sources using OLED technology may lead to important new applications in areas such as biology,^[^ ^2^, ^3^, ^4^, ^5^, ^6^, ^7^ ^]^ medicine,^[^ ^8^, ^9^, ^10^, ^11^ ^]^ and communication.^[^ ^12^, ^13^, ^14^, ^15^ ^]^

Given growing global concerns about the sustainable and energetically efficient operation of electronics—many of them with limited battery capacity—optimizing the efficiency with which OLEDs convert electrical energy into light has been a focus of intense research over the last three decades.^[^ ^16^ ^]^ This has led to the development of OLEDs with overall charge‐to‐photon conversion efficiency, also known as external quantum efficiency (EQE), in excess of 35%^[^ ^17^, ^18^, ^19^, ^20^, ^21^, ^22^, ^23^, ^24^ ^]^ and to power efficacies exceeding those of fluorescent tubes and conventional inorganic LEDs.^[^ ^25^ ^]^ In the most efficient OLEDs, the efficiency of the internal light generation process is generally understood to be nearly 100%, that is, every electron passing through the device generates a photon. However, the efficiency of extraction of the generated photons from the device (typically referred to as “outcoupling efficiency”) remains a major bottleneck to the overall EQE, with 70% to 80% of the generated photons remaining trapped within the OLED in many state‐of‐the‐art devices.

**Figure 1:**
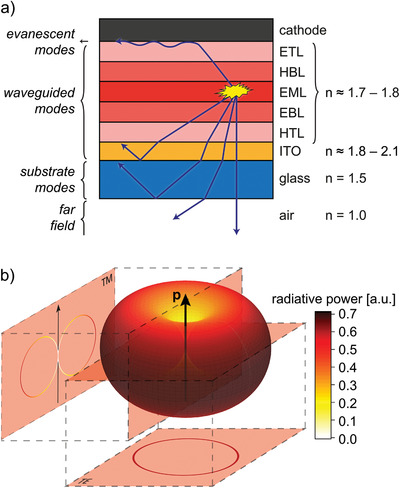
a) Basic structure of an OLED stack, with layers of different materials and refractive indices: cathode, electron‐transport layer (ETL), hole‐blocking layer (HBL), emissive layer (EML), electron‐blocking layer (EBL), hole‐transport layer (HTL), transparent anode—commonly made of indium tin oxide (ITO)—and transparent substrate (glass). The arrows denote the possible trajectories of photons generated through radiative relaxation of an exciton localized on an emissive molecule. Only a fraction of photons ultimately leaves the device. b) Spatial distribution of the radiation from a classical Hertzian dipole. The maximum of the distribution is directed perpendicular to the dipole.

The basic structure of an OLED is schematically illustrated in **Figure** **1**a. It comprises one or multiple layers of organic semiconductors with a total thickness in the range of 80–200 nm that are sandwiched between two electrodes, at least one of which is partially transparent. Positive and negative charges (referred to as holes and electrons, respectively) are injected into the organic semiconductor layers from the electrodes and travel toward each other to recombine within the emissive layer (EML), usually located approximately in the middle of the device stack. There, they form bound electron–hole pairs (excitons) that involve electronically excited states of one or more molecules. Each exciton can relax back to the ground state through the emission of a photon. The generated photons then interact with the layered thin‐film architecture of the OLED through reflection, refraction, thin‐film interference, and absorption. Eventually, some photons escape from the device to provide useful light. The fraction of photons emitted from an OLED over the total number of photons generated internally is influenced by a number of factors, including the thicknesses and refractive indices of the layers forming the OLED, and also by the direction of the photon generated during exciton relaxation.^[^ ^26^, ^27^ ^]^ The latter depends on the orientation of the emissive molecules (emitters) on which the excitons are localized when the relaxation takes place. More specifically, photons are predominantly emitted perpendicular to the transition dipole moment (TDM) of each emitter and the emission probability gradually falls off for angles closer to the axis of the TDM—that is, the intensity closely follows the “donut”‐shaped distribution of a classical Hertzian dipole (Figure 1b). As a result, the outcoupling efficiency of an OLED can be increased significantly if emitters are oriented such that TDMs align horizontally with respect to the plane of the device.

Understanding the impact of emitter orientation on outcoupling efficiency in detail, developing robust methods for measuring emitter orientation within the EML, and identifying the factors that influence orientation in the EMLs of state‐of‐the‐art OLEDs have recently become topics of intense research.^[^ ^28^, ^29^, ^30^, ^31^, ^32^, ^33^ ^]^ While the polymers used in many solution‐processed OLEDs have for a long time been known to show a high degree of orientation parallel to the plane of the film, the low‐molecular‐weight emitters (referred to as “small molecules” by the community) were originally assumed to be randomly oriented. However, over the last decade, this has been debunked and by now there are many examples of small‐molecule‐based OLEDs that show enhanced light‐outcoupling efficiency owing to preferential horizontal orientation of the emitter TDM. In fact, an ever‐increasing number of reports document how the molecular orientation of the emitter contributes to improved OLED efficiency.

**Figure 2:**
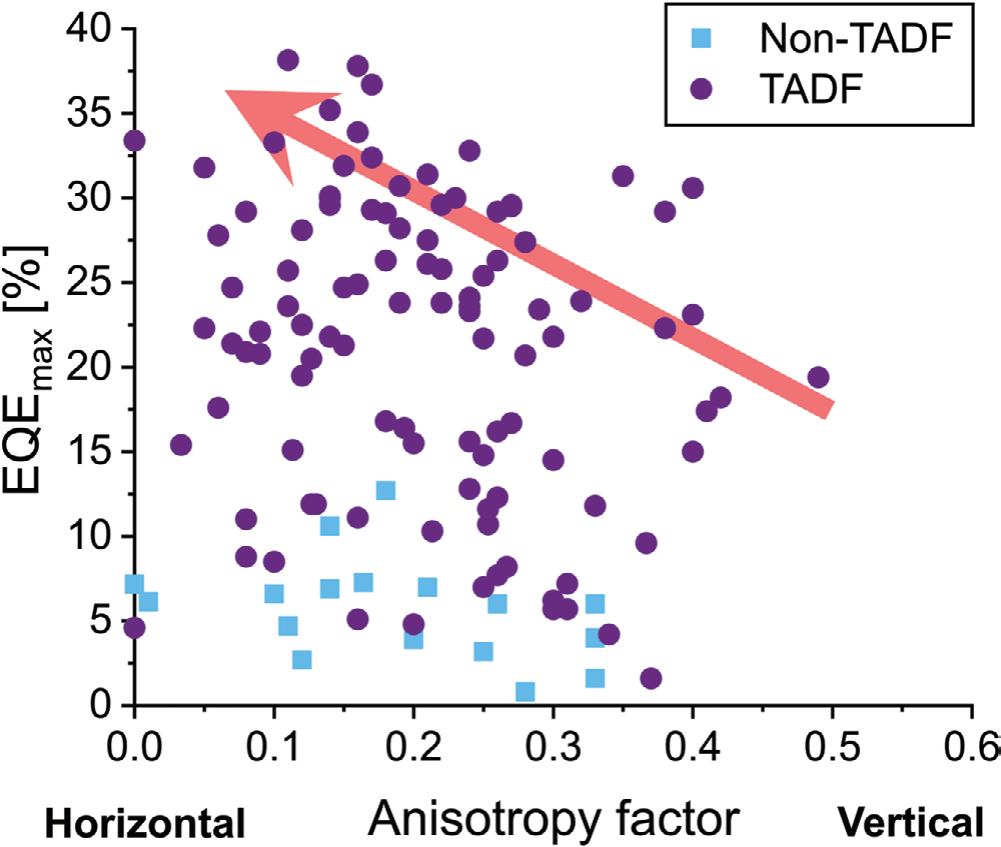
EQE_max_ as a function of emitter orientation in OLEDs based on purely organic emitters. The figure was made using data from 53 separate reports that include EQE_max_ and emitter orientation data on 124 materials systems.^[^ ^20^, ^22^, ^23^, ^24^, ^37^, ^38^, ^39^, ^40^, ^41^, ^42^, ^43^, ^44^, ^45^, ^46^, ^47^, ^48^, ^49^, ^50^, ^51^, ^52^, ^53^, ^54^, ^55^, ^56^, ^57^, ^58^, ^59^, ^60^, ^61^, ^62^, ^63^, ^64^, ^65^, ^66^, ^67^, ^68^, ^69^, ^70^, ^71^, ^72^, ^73^, ^74^, ^75^, ^76^, ^77^, ^78^, ^79^, ^80^, ^81^, ^82^, ^83^, ^84^, ^85^ ^]^ While there is no correlation between EQE_max_ and anisotropy across all data, it is clear that the highest efficiency in TADF‐ and non‐TADF‐based systems, respectively, is reached at low anisotropy factors.

Over the better part of the last decade, most of the molecular orientation research was centered on elucidating the factors that determine the orientation of organometallic phosphorescent emitters (mainly Ir‐ and Pt‐based compounds).^[^ ^29^, ^30^ ^]^ This was mostly due to the use of this class of emitters in current state‐of‐the‐art, commercial devices and thus their popularity within the research community. However, with the advent of comparably efficient organic thermally activated delayed fluorescence (TADF) OLEDs, focus has turned to identifying design guidelines for achieving horizontally oriented, fully organic molecules. This has led to the identification of key parameters that drive the molecular orientation of emitters in TADF‐based OLEDs, which has largely contributed to devices achieving maximum EQEs (EQE_max_) upward of 35% (see **Figure** **2**).^[^ ^20^, ^22^, ^23^ ^]^ For example, it has been long established—largely due to the extensive work by Yokoyama et al.—that molecules with higher aspect ratios tend to orient more horizontally in a film via the maximization of weak, intermolecular van der Waals interactions.^[^ ^28^ ^]^ However, while the argument of molecular aspect ratio is often used in the literature, aspect ratios are rarely quantified, making it difficult to cross‐compare different reports. Additionally, the glass‐transition temperature (*T*_g_) of the films that constitute the EML has also been identified to play a key role in arresting the molecular surface diffusion during film growth,^[^ ^34^, ^35^ ^]^ which contributes to improving the molecular alignment of emitters.^[^ ^36^ ^]^ However, there are very few systematic studies on the influence of *T*_g_ on the orientation of emitters. Consequently, to date the true impact of these factors, as well as clear design guidelines for achieving horizontal molecular orientation, remain elusive.

The identification of key parameters that can inform the long‐sought guidelines is not straightforward. Qualitative descriptors such as linearity or planarity do not have a well‐established, quantitative definition. Moreover, even in systematic studies of structurally related molecules, variations in linearity and planarity cannot be readily decoupled from variations of other parameters such as molecular weight (MW) and size. Additionally, each of these parameters influences the *T*_g_ of the films, rendering the problem of identifying key parameters even more complex. Over the last few years, a substantial body of literature has been published that reports on the orientation of a wide variety of fluorescent and TADF emitters from a range of systems. Cross‐comparing the available orientation data from these studies may well provide relevant insights beyond the conclusions that can be drawn from individual studies.

Thus, in addition to providing a detailed review of the current research on emitter orientation in organic fluorescent and TADF OLEDs, we perform a meta‐analysis of published orientation data. We combine this with extensive modeling of molecular geometry for published emitter and host molecules to identify the most influential molecular parameters that drive horizontal orientation of emitters in OLEDs. We begin by briefly reviewing the impact of TDM orientation on outcoupling efficiency of devices. We then provide an overview of the state of the art in emitter orientation studies for fluorescent and TADF OLEDs, noting how the key parameters have been identified so far, and reviewing new, promising strategies for achieving horizontally oriented emitters. The main body of this review is devoted to presenting the results of a statistical analysis of the available data on orientation studies of fully organic emitters in vacuum‐processed EMLs for OLEDs. This includes: i) the definition of a set of possible influential parameters and, where required, modeling of these parameters using density‐functional theory (DFT) calculations, ii) a study of the correlation of each of these parameters with molecular orientation data and with the other parameters of the set, and iii) the conclusions that we drew from those correlations. We close with a general summary and an outlook on possible future work.

## 2 Influence of the Orientation of the Emitter Transition Dipole Moment on the Outcoupling Efficiency of OLEDs

The EQE of an OLED can be expressed as the product of the internal quantum efficiency (IQE)—that is, the total internal charge‐to‐photon conversion ratio—and the outcoupling efficiency, η_out_—that is, the fraction of photons that are extracted from the device. In turn, the IQE can be expressed as the product of three factors that depend on the electronic properties of the device and on the emitter molecules used, yielding

(1) $$\begin{array}{c} \text{EQE}\;\;=\;\;\gamma \xi_{e}\;\Phi_{\text{eff}}\;\eta_{\text{out}} \end{array}$$

where γ is the charge balance, ξ_e_ is the spin factor, and Φ_eff_ is the effective radiative quantum efficiency of the emitter molecules in the device. The IQE and the factors influencing it, such as efficiency roll‐off through exciton–exciton or exciton–polaron annihilation, have been reviewed in detail elsewhere and thus will not be discussed here.^[^ ^86^, ^87^ ^]^ η_out_ can be expressed as the ratio of the radiated optical power that can escape the device (*U*) to the total power radiated by the emitting molecules inside the OLED (*F*):

(2) $$\begin{array}{c} \eta_{\text{out}}=\frac{U}{F} \end{array}$$

**Figure 3:**
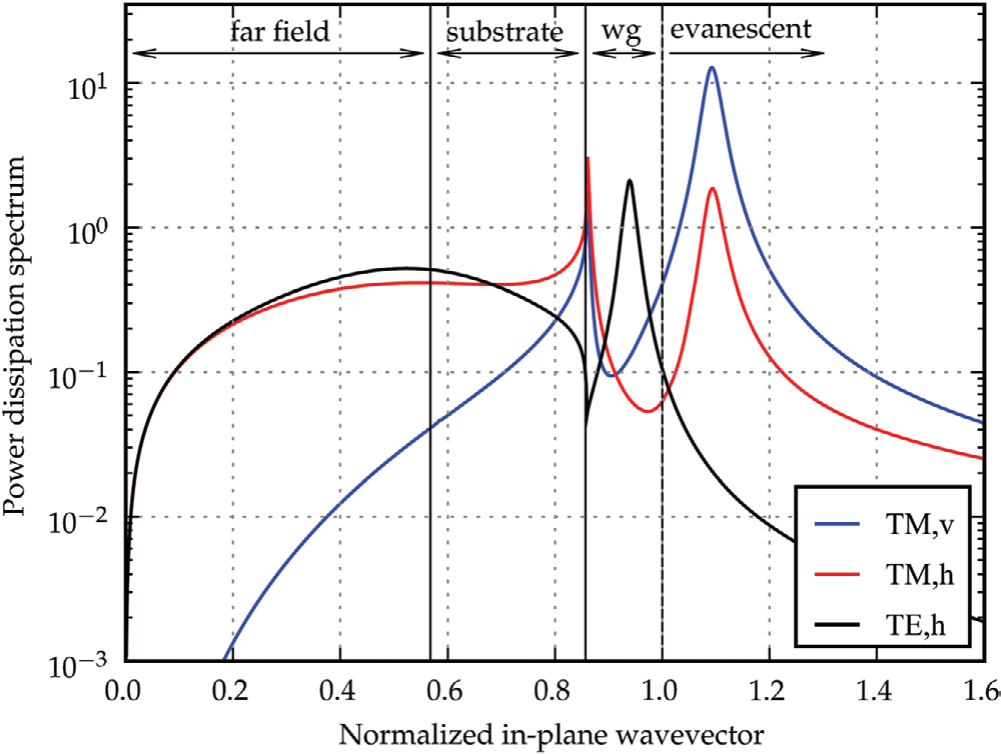
Representative power dissipation spectra for a red bottom‐emitting OLED at λ = 610 nm. The in‐plane wavevector is normalized with respect to propagation in the EML. The spectra are shown for vertical dipoles (TM,v) and for horizontal dipoles which couple to both TM and TE cavity modes (TM,h and TE,h, respectively). Adapted with permission.^[^ ^91^ ^]^ Copyright 2010, Society of Photo‐Optical Instrumentation Engineers (SPIE).

In the classical dipole approximation,^[^ ^26^, ^88^, ^89^, ^90^ ^]^ which is the basis for most state‐of‐the‐art molecular orientation studies, the total radiated power from an ensemble of individual, non‐interacting dipoles can be expressed as

(3) $$\left\langle p\right\rangle =\left\langle p_{x}+p_{y}+p_{z}\right\rangle$$

where the brackets indicate averaging over the ensemble of individual dipoles. The spectral power density (*K*) radiated by the ensemble can in turn be separated into its polarization components: one transverse electric (TE) and two transverse magnetic (TM) components, which can be conveniently labelled as vertical (v) or horizontal (h) according to their orientation with respect to the plane of the film. In this way, TE,h, TM,h, and TM,v are equivalent to the *x*, *y*, and *z* coordinates in Equation (3), respectively. *K* can then be expressed in terms of the emission spectrum of the dipoles, the spatial distribution of their radiation, and their orientation with respect to the plane of the film as

(4) $$\begin{array}{c} K\left(\lambda ,u,\theta \right)=\left[K_{\text{TM},h}\left(\lambda ,u\right)+K_{\text{TE},h}\left(\lambda ,u\right)\right]\left\langle \sin^{2}\theta \right\rangle +K_{\text{TM},v}\left(\lambda ,u\right)\left\langle \cos^{2}\theta \right\rangle \end{array}$$

where λ denotes the wavelength of the emission, *u* denotes the normalized in‐plane component of the wavevector (direction of the emission),^[^ ^27^ ^]^ and θ is the angle of the individual dipoles with respect to the normal of the film. It can be seen from **Figure** **3** that the three components of *K* contribute differently to the fraction of the power dissipation spectrum that is radiated into the far field. In particular, this fraction is much higher for the TM,h and TE,h components than for the TM,v component of *K*.

*U* and *F* (see Equation (2)) can then be obtained from the integration of *K* over *u* and λ as

(5) $$\begin{array}{c} U\left(\theta \right)=\int_{\lambda }\int_{u_{\text{crit}}\left(\lambda \right)}^{0}s\left(\lambda \right)\;K\left(\lambda ,u,\theta \right)\;du^{2}d\lambda \end{array}$$

and

(6) $$\begin{array}{c} F\;\left(\theta \right)=\int_{\lambda }\int_{\infty }^{0}s\left(\lambda \right)\;K\left(\lambda ,u,\theta \right)\;du^{2}d\lambda \end{array}$$

where *s*(λ) is the normalized intrinsic emission spectrum of the emitters ($\int_{\lambda }s(\lambda )\;d\lambda \;=\;1$) and *u*_crit_(λ) is the value of *u* at which total internal reflection occurs for each wavelength, meaning that emission above *u*_crit_ is no longer transmitted into the far field but trapped inside the device. From Equations ((2), (4)–6) it is clear that η_out_ depends strongly on the orientation of ***p***, with η_out_ being maximal for θ = 90 ° (horizontal orientation). Remarkably, η_out_ can be up to 50% higher for an ensemble of completely horizontal emitter dipoles than for a randomly distributed (isotropic) ensemble.^[^ ^92^ ^]^

## 3 Quantifying the Orientation of the Emitter Transition Dipole

The experimental quantification of θ can be done through various experimental methods that have been reviewed elsewhere.^[^ ^29^, ^30^ ^]^ From these, the two most common ones are variable‐angle spectroscopic ellipsometry (VASE) and angle‐resolved luminescence spectroscopy (ARLS).^[^ ^93^, ^94^, ^95^ ^]^ ARLS can refer to electroluminescence, if one measures the emission from an OLED, or photoluminescence, if this quantity is measured from an optically excited EML.^[^ ^96^, ^97^ ^]^ Importantly, VASE and ARLS provide information about the orientation of different transition dipoles in the EML. A single emitter molecule can have more than one TDM of absorption and emission, and their orientation is not necessarily the same, depending on the states involved in the transition.^[^ ^41^, ^77^, ^98^, ^99^ ^]^ It is particularly important to keep this in mind when comparing data from VASE and ARLS measurements. While VASE probes the average TDM of absorption of the dominantly absorbing species in the film, ARLS probes the average TDM of emission of the dominantly emitting species. The orientation of these average dipoles depends on the electronic states that are involved in each transition, which are not necessarily the same. In addition, in host–guest EMLs, the transition dipole of the guest molecules is frequently undetectable by VASE as it is overshadowed by the low energy tail of the host absorption. Thus, care must be taken when relating the measured orientation of the transition dipole to the different transitions that can take place.

The development of different experimental methods for measuring the orientation of the TDM of emitters has also led to different parameters being used for this quantification. ARLS studies commonly, but not exclusively, report the fraction of vertically oriented emission dipoles through the anisotropy factor:

(7) $$\begin{array}{c} a\equiv \left\langle \cos^{2}\theta \right\rangle \end{array}$$

or through the fraction of horizontally oriented dipoles:

(8) $$\begin{array}{c} \Theta \equiv \left\langle \sin^{2}\theta \right\rangle \end{array}$$

By contrast, results from VASE measurements are more commonly reported in terms of the order parameter:

(9) $$\begin{array}{c} S\equiv \frac{3\left\langle \cos^{2}\theta \right\rangle -1}{2} \end{array}$$

The details about how to obtain *a*, *Θ*, and *S* from ARLS and VASE measurements can be found elsewhere.^[^ ^28^, ^29^, ^95^ ^]^ Conversion from one parameter to another can be done using Equations (7–9).

It is important to note that the advancement of computational modeling techniques has recently made it possible to model the orientation of vacuum‐deposited organic molecules through DFT and molecular dynamics calculations. These have been recently reviewed elsewhere,^[^ ^31^, ^35^, ^100^ ^]^ so here we have focused on results from experimental measurements. The vast majority of reports included in this review used ARLS. Thus, we henceforth use the term TDM referring to the emission dipole moment of the molecules. However, for neat film systems, we have included reports that used VASE for measuring the molecular orientation due to the limited availability of ARLS data for these systems. For the purposes of our analysis, we have assumed similar orientations of absorption and emission TDMs in these few molecules. For simplicity, we have converted all literature values to their equivalent value in terms of *a*. In this framework, *a* = 0 represents perfectly horizontal orientation of the average TDM and *a* = 1 represents perfectly vertical orientation. Importantly, an isotropic distribution of dipole orientations would yield *a* = 1/3.^[^ ^95^ ^]^ It is also important to point out that obtaining accurate values of either of the parameters mentioned above requires accurate measurements of the optical constants of the film (or of the multiple layers in the OLED stack if orientation is measured in a complete OLED) and, in the case of ARLS, of its (intrinsic) photoluminescence or electroluminescence spectrum. Additionally, both VASE and ARLS require careful optical modeling. These factors can render the accuracy in the calculation of the TDM orientation hard to estimate and the associated error is often not reported. Based on our own experience, the error when obtaining the anisotropy factor via ARLS is usually between ±0.01 and ±0.02 in units of *a*, but it can be higher if the optical constants are not accurately measured, or if the sample has poor emission. Thus, we advise that claims of “perfect” horizontal orientation should be taken with caution.

## 4 Currently Pursued Strategies for Achieving Horizontal Orientation

During the formation of vacuum‐deposited films, the evaporated molecules land on the substrate or on other previously deposited molecules. Then, they diffuse over the surface until they are buried by other incoming evaporated molecules, after which they remain trapped, potentially in a metastable state.^[^ ^34^, ^35^ ^]^ Thus, it can be expected that the orientation of the molecules in the film depends on a set of variables directly related to this kinetically controlled process. As we mentioned above, finding these key variables remains an open problem. So far, three main strategies are discussed in the literature to control the orientation of organic emitters in vacuum‐processed films for OLEDs: optimizing the molecular shape, selecting host molecules with suitable *T*_g_ in co‐evaporated systems, and tuning the temperature of the substrate during the deposition of the film.

### 4.1 Molecular Shape

**Figure 4:**
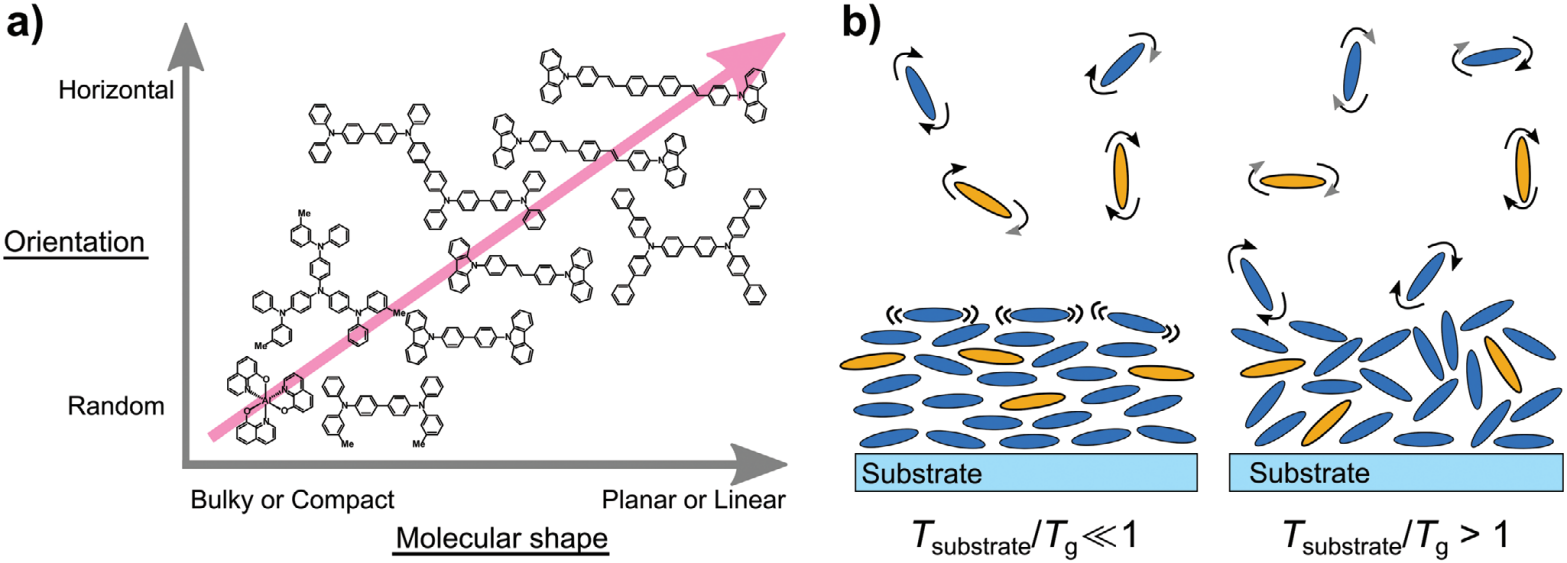
a) Geometrical factors promoting horizontal alignment of organic molecules in vacuum‐deposited films. Planar or linear molecules with extended configurations have been observed to adopt a higher degree of horizontal orientation in thin films. Adapted with permission.^[^ ^28^ ^]^ Copyright 2011, The Royal Society of Chemistry. b) Influence of the *T*_g_ and the temperature of the substrate (*T*_substrate_) on the orientation of organic emitters in host–guest systems. Deposition of the molecules at *T*_substrate_/*T*_g_ ≪ 1 has been observed to promote horizontal alignment. By contrast, deposition at *T*_substrate_/*T*_g_ > 1 has been observed to promote randomization of molecular orientation.

Yokoyama et al. extensively studied the molecular orientation of doped and neat films of organic emitters, as well as of organic hole‐ and electron‐transport materials. They found that more planar and more linear molecules tend to align more horizontally in neat films.^[^ ^24^, ^28^, ^67^, ^76^, ^93^, ^101^, ^102^, ^103^, ^104^ ^]^ This trend is illustrated in **Figure** **4**a. Similar results have been obtained by a number of other groups in both neat films and host–guest systems.^[^ ^20^, ^23^, ^38^, ^40^, ^41^, ^45^, ^46^, ^47^, ^48^, ^49^, ^52^, ^53^, ^54^, ^76^, ^77^, ^105^ ^]^ From these studies, it has become a widely accepted dogma in the field that more extended molecules promote horizontal orientation of emitters, thereby increasing the outcoupling efficiency of devices. This improved orientation is often attributed to an increased aspect ratio of the molecules (higher linearity or planarity). However, it is important to note that in most of these reports the increase in linearity or planarity is achieved by adding segments to a parent molecular structure. Thus, this change cannot be decoupled from an increase in the size and the MW of the emitter.

### 4.2 Glass‐Transition Temperature

Organic materials with higher *T*_g_ are less prone to surface diffusion during vacuum deposition.^[^ ^34^ ^]^ Mayr et al. studied the influence of the *T*_g_ of the host materials on the orientation of coumarin 6, a fluorescent emitter with relatively low MW.^[^ ^36^ ^]^ They found that the horizontal alignment of the TDM of this compound was strongly correlated to the *T*_g_ of the host in which it was deposited, going from a completely isotropic orientation in hosts with low *T*_g_ to preferentially horizontal orientation in hosts with higher *T*_g_. The authors ascribed this to a decrease in surface diffusion during film formation when hosts with higher *T*_g_ were used (Figure 4b). This trend has also been observed in other molecules with relatively low MW (2CzTPN and 4CzTPN) studied by Tanaka et al.^[^ ^81^ ^]^ and, more recently, in the similarly light molecule DMAC‐TRZ studied by Naqvi et al.^[^ ^106^ ^]^ It is worth noting that the studies by Mayr et al. and by Naqvi et al. are the only ones in which this influence has been shown for a large set of hosts spanning a wide range of *T*_g_ (62–176 °C). What is more, Naqvi et al. also found that the *T*_g_ of the host has only a very modest influence on the orientation of the larger and heavier molecule ICzTRZ. This suggests that the *T*_g_ of the host molecules is only relevant for small, low‐MW emitters.

### 4.3 Substrate Temperature

An alternative way of reducing the surface diffusion of molecules during film formation and thus potentially improving their horizontal alignment is to change the temperature of the substrate during the deposition process.^[^ ^107^, ^108^, ^109^ ^]^ Deposition at low temperatures (e.g., at 200 K) has helped to improve horizontal alignment of TDMs in neat and doped films, achieving near‐perfect horizontal dipole orientation in some cases.^[^ ^39^, ^40^, ^110^ ^]^ By contrast, deposition at a temperature close to the *T*_g_ of the host through active heating of the substrate yielded an isotropic distribution of TDM orientations.^[^ ^40^, ^110^ ^]^ These reports have been reviewed in detail elsewhere.^[^ ^29^, ^30^, ^31^, ^32^ ^]^

Of the three strategies, the one that has been invoked most frequently is the improvement of the molecular shape. However, modifying the geometry of model emitters while preserving or improving their photophysical properties has presented challenges for the design of highly efficient, highly oriented emitters. This has been the case particularly for TADF and deep‐blue, fluorescent molecules due to the constraints that their photophysics pose on their chemical architectures. In the next section, we review these challenges and the recent research avenues that have been pursued to tackle them.

## 5 Recent Demonstrations of Horizontal Orientation in New TADF and Deep‐Blue, Fluorescent Emitters

Preferential alignment of the main axis or main plane of a molecule with the substrate does not necessarily guarantee the same alignment of the TDM. This is particularly relevant for TADF molecules, which are commonly composed of donor (D) and acceptor (A) moieties linked together in a twisted conformation that results in a decrease of the singlet–triplet energy gap (Δ*E*_ST_). In this kind of structure, the TDM points in the direction that connects these two moieties. If the molecule is composed of only one D and one A, the direction of the TDM generally coincides with its long axis; however, this is not always the case. In some molecules, this twisted conformation may promote an offset angle between the TDM and either (or both) the longer axes of the molecule.^[^ ^44^ ^]^ Thus, an improved horizontal orientation of the molecular plane could induce an out‐of‐plane, even preferentially vertical alignment of the TDM, thereby reducing rather than increasing the outcoupling efficiency. This can also be the case in molecules composed of more than one D and one A moiety. It is therefore important to be mindful of the direction of the TDM with respect to the structure of the molecule when working to improve the alignment of emitters.^[^ ^44^, ^57^ ^]^

**Figure 5:**
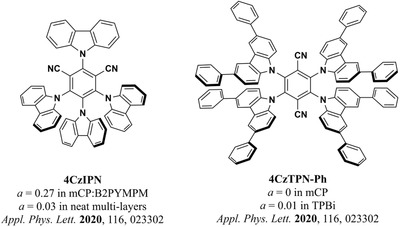
Two examples of carbazole‐based, bulky, disk‐shaped TADF emitters reported by Adachi et al.^[^ ^79^, ^81^ ^]^

The interplay between the importance of a twisted donor–acceptor architecture in TADF molecules and the search for geometries that favor horizontal alignment during vapor deposition has led to the development of new strategies in molecular design. A recent approach for improving horizontal alignment of TADF emitters explored by Adachi et al. actually makes use of their twisted architectures and employs a bulky, disk‐shaped molecular design with many carbazole‐based donors around a central phthalonitrile acceptor (**Figure** **5**).^[^ ^71^, ^79^, ^81^, ^111^ ^]^ For example, Sun et al. found that 4CzIPN has slightly preferential horizontal orientation (*a* = 0.27) when doped at 5 wt% into a co‐host matrix mCP:B2PYMPM (1:1).^[^ ^79^ ^]^ The authors ascribed this to the fact that the TDM of 4CzIPN is aligned parallel to the *xy* plane of the molecule and argued that parallel stacking of the molecule with respect to the substrate is promoted by 4CzIPN having “a flat, planar structure,” even though the planarity of 4CzIPN was not quantified in the study. In a later report, Hasegawa et al. deposited mono‐ and multi‐layers of the same emitter (4CzIPN) on gold and glass substrates and found an almost perfect horizontal orientation of the emitter (*a* = 0.03).^[^ ^111^ ^]^ They achieved this by controlling the deposition rate to grow just 1 monolayer per 1000 s. Unfortunately, so far it has not been possible to preserve this orientation of 4CzIPN in thicker films deposited at higher rates, as would commonly be the case in OLED fabrication.

Recently, Tanaka et al. studied the orientation of polar and non‐polar bulky disk‐shaped TADF molecules in hosts with different *T*_g_ and polarity.^[^ ^81^ ^]^ Remarkably, many of the molecules in this report achieved a high degree of horizontal orientation (*a* < 0.10) without having particularly high aspect ratios, as we will discuss below. One of them (4CzTPN‐Ph) even achieved a perfectly horizontal orientation within the precision of the measurement. Although the PLQY of this emitter was rather low in the doped CBP film (16%), the authors showed that adding bulky substituents to carbazole‐based TADF molecules can be a promising design strategy for achieving high outcoupling efficiencies in OLEDs. More recently, Cui et al. used another bulky, carbazole‐based emitter (5CzTRZ, Figure 5) in a device that achieved an EQE_max_ of 29.3%.^[^ ^71^ ^]^ This high EQE_max_ value was partly due to the preferentially horizontal orientation of 5CzTRZ in the doped mCBP film (*a* = 0.17).

**Figure 6:**
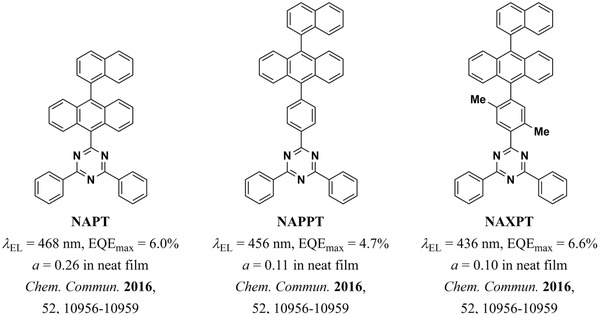
TADF molecules reported by Kim et al.^[^ ^38^ ^]^ The inclusion of bridging units in NAPPT and NAXPT increased their aspect ratio with respect to NAPT, thereby improving their horizontal orientation. Additionally, the twisting of the xylene ring due to steric hindrance by the methyl groups led to deeper blue emission.

Still, the most common strategy in terms of molecular design for enhancing the outcoupling efficiency in TADF devices is to employ architectures that lead to larger aspect ratios. A particularly important challenge in this regard is how to improve the aspect ratio of these molecules without increasing their conjugation length. Greater conjugation can lead to a redshift of the emission, which is usually undesired, particularly for blue and deep‐blue emitters. Thus, increasing the aspect ratio of this kind of molecule while preserving—or indeed improving—their photophysical properties has been the focus of a number of groups over the last five years. One strategy is the inclusion of a twisted bridging unit between the D and A moieties, usually a phenyl ring. This has been shown to reduce the conjugation of the molecules and favor horizontal alignment of the TDM by increasing the aspect ratio of the molecules. For example, Kim et al. reported the inclusion of a phenyl or a xylene ring between the D and A units of modified versions of the molecule NAPT (**Figure** **6**).^[^ ^38^ ^]^ These bridging moieties increased the aspect ratio of the modified molecules NAPPT and NAXPT, thereby improving their horizontal orientation. Additionally, the enhanced twisting of the phenyl and xylene bridges resulted in progressively smaller conjugation lengths of the two emitters and pushed the emission of the molecule deeper into the blue.

**Figure 7:**
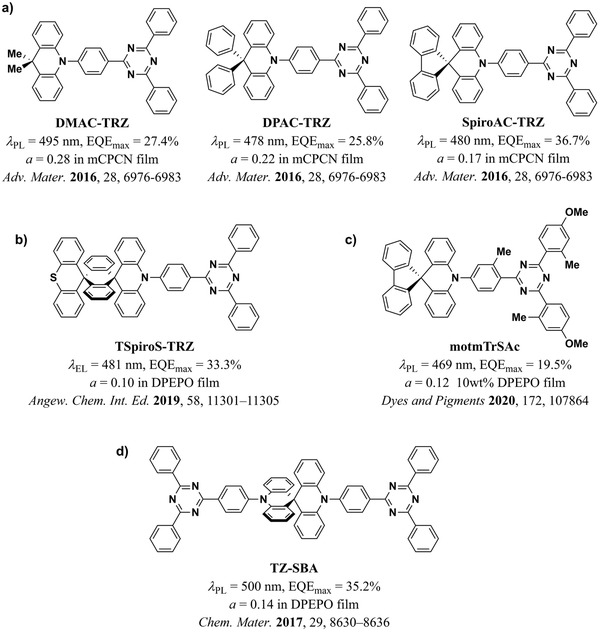
a) Modifications to the DMAC‐TRZ molecule reported by Lin et al.^[^ ^20^ ^]^ The higher aspect ratio of SpiroAC‐TRZ contributed to its more horizontal orientation and to the higher EQE_max_ in the device. b) TspiroS‐TRZ and c) motmTrSAc, two further modifications to SpiroAC‐TRZ reported by Li et al.^[^ ^48^ ^]^ and by Lee et al.,^[^ ^49^ ^]^ respectively. d) TZ‐SBA, an A–D–D–A structure based on the AC and TRZ moieties, reported by Liu et al.^[^ ^24^ ^]^

An extension of this strategy is based on the addition of further rigid, twisted moieties that increase the aspect ratio of the molecules and reduce their conjugation length, which leads to a localization of their HOMO and LUMO on the D and A moieties, respectively. Indeed, with DPAC‐TRZ and SpiroAC‐TRZ, Lin et al. reported two analogues of the previously reported molecule DMAC‐TRZ based on this concept (**Figure** **7**a).^[^ ^20^ ^]^ The high PLQY and improved horizontal orientation (*a* = 0.17) of SpiroAC‐TRZ led to a sky‐blue OLED with a remarkable EQE_max_ of 37%. This strategy was further exploited by Li et al.,^[^ ^48^ ^]^ who further increased the rigidity and length of the molecule SpiroAC‐TRZ by adding an additional orthogonal donor moiety (Figure 7b). The resulting emitter TspiroS‐TRZ achieved *a* = 0.10 and blue OLEDs based on it achieved 33% EQE_max_. Further structural modifications of SpiroAC‐TRZ were recently reported by Lee et al.^[^ ^49^ ^]^ In this case, the authors increased the aspect ratio of the new molecules by adding substituents to the ato an improved horizontal orientation of motmTrSAc (*a* = 0.12) compared to SpiroAC‐TRZ (*a* = 0.22) when doped in a DPEPO host. However, this improvement in orientation did not translate into an improvement in the EQE_max_ due to the reduced PLQY of motmTrSAc (PLQY = 51%, EQE_max_ = 19.5%).

**Figure 8:**
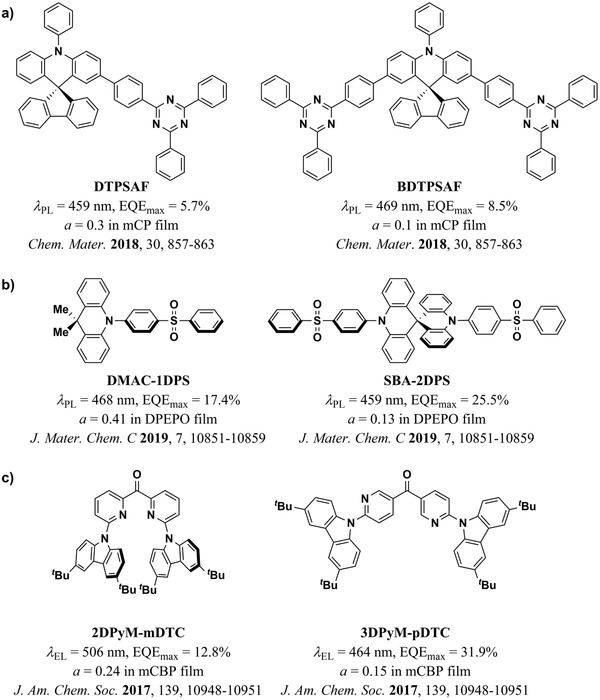
Examples of molecules with increased aspect ratios via symmetrically placed D and A units. a) DTPSAF and BDTPSAF.^[^ ^105^ ^]^ b) DMAC‐1DPS and SBA‐2DPS.^[^ ^53^ ^]^ c) 2DPyM‐mDTC and 3DPyM‐pDTC.^[^ ^45^ ^]^

A different approach for increasing the aspect ratio of TADF molecules consists of the adoption of an A–D–A or D–A–D architecture. For example, Woo et al. reported a large increase in the horizontal orientation of the A–D–A molecule BDTPSAF (*a* = 0.10) when compared to its D–A counterpart DTPSAF (*a* = 0.30) (**Figure** **8**a).^[^ ^105^ ^]^ This allowed to achieve nearly 10% EQE_max_ in deep‐blue TADF OLEDs. A similar concept was explored by Liu et al.^[^ ^24^ ^]^ (Figure 7d) and later by Zeng et al.^[^ ^53^ ^]^ (Figure 8b) in long, stick‐like molecules with two orthogonal pairs of D–A units. In both cases, the two D moieties constituted a spiro biacridine (SBA) center. The two A units were then symmetrically placed around the SBA. This approach significantly improved the orientation of the A–D–D–A molecules with respect to their D–A analogues, enabling Liu et al. and Zeng et al. to make devices with EQE_max_ of 35.2% and 25.5%, respectively. Rajamalli et al. also explored the influence of the position of two D moieties symmetrically placed around a central A unit (Figure 8c).^[^ ^45^ ^]^ They showed that the more extended isomer 3DPyM‐*m*DTC adopted a higher degree of horizontal orientation (*a* = 0.15) with respect to the more globular 2DPyM‐*m*DTC (*a* = 0.24). Additionally, the former achieved a blueshifted emission and a higher EQE_max_ (31.9%) than the latter (12.8%) when doped in mCBP.

**Figure 9:**
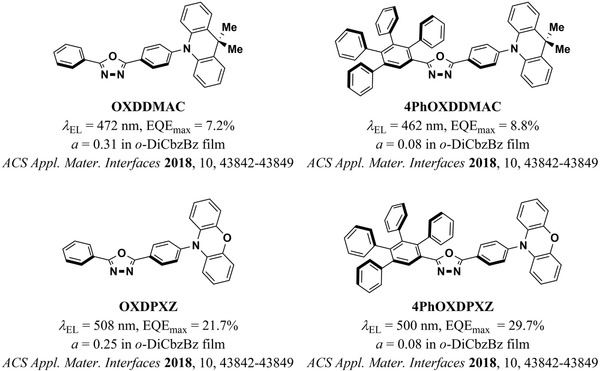
Molecules with increased aspect ratios by the addition of multiple sterically hindered phenyl rings.^[^ ^58^ ^]^ The orientation of 4PhOXDDMAC and 4PhOXDPXZ is largely improved with respect to that of OXDDMAC and OXDPXZ.

Lee et al. reported another strategy for increasing the aspect ratio of TADF molecules without increasing their conjugation length.^[^ ^58^ ^]^ This consists of the addition of an increasing number of sterically hindered phenyl groups to one of the ends of the OXDDMAC and OXDPXZ molecules (**Figure** **9**). The authors showed that the modified tetraphenylated versions of these molecules adopted a more horizontal orientation than the unmodified ones in various hosts. Remarkably, they achieved *a* = 0.08 and an EQE_max_ of 29.2% when doping 4PhOXDPXZ into the host *o*‐DiCbzBz.

In summary, several different strategies have been identified for improving the horizontal alignment of the TDM of organic emitter molecules and these have been exploited in several families of emitter systems. We close this section by highlighting challenges that may still hinder the identification of clear design guidelines for this kind of molecules. First, in the many studies that have reported improved horizontal alignment of molecules with higher aspect ratios compared to other molecules with similar structures, claims on linearity and planarity are often evoked but rarely quantified.^[^ ^20^, ^24^, ^38^, ^39^, ^40^, ^41^, ^45^, ^46^, ^47^, ^48^, ^49^, ^52^, ^53^, ^54^, ^57^, ^60^, ^65^, ^66^, ^67^, ^75^, ^93^, ^112^ ^]^ As we mentioned above, the real impact of an increase in the aspect ratio of molecules is difficult to assess because the studies are often based on the addition of moieties to reference molecules. Therefore, the reported increases in the linearity or planarity of the molecules are accompanied by increases in MW. Furthermore, changes in the aspect ratio and MW of the components of a film can also alter the *T*_g_ and change the diffusion process of the molecules during film formation.^[^ ^113^, ^114^ ^]^ Unfortunately, the *T*_g_ of the emitter films (either neat or doped) is rarely reported. Thus, it can be hard to distinguish whether it is the actual aspect ratio of the molecules, their higher MW, the associated increase in *T*_g_, or a combination of these parameters that is responsible for promoting the more horizontal TDM alignment of the emitter in the film. From a materials science perspective, it is important to establish the relation between these parameters and their influence on the surface‐diffusion process. This could provide useful a priori information about the possible molecular orientation of newly designed emitters. In the next part of this review, we analyze the influence of the parameters reviewed above on the orientation of organic emitters. This allows us to identify the parameters that have the strongest impact on driving horizontal alignment of the TDMs of these molecules.

## 6 Methodology for Meta‐Analysis of Literature Data and DFT Modeling

We first built a database documenting the properties of 130 different fluorescent and TADF emitters, gathered from 57 previous studies. The orientation of these 130 emitters was reported in neat or doped films, sometimes involving different host–guest combinations or doping concentrations. This yielded a total of 203 different “systems,” each composed of a unique combination of host and emitter. Each system constitutes one entry in our database, which is publicly available (see Data Availability Statement below). The database was populated by taking the following reported parameters from the respective papers: the anisotropy factor (or equivalent parameter), the host and emitter *T*_g_ (where reported), the doping concentration (for doped films), the molecular structure, and the EQE_max_ of the devices using the system (where available). For consistency, the rest of the parameters were extracted from computational simulations performed in‐house, as described below.

### 6.1 Definition of Parameters

For each molecule in our database, a DFT ground state optimization was first performed using the B3LYP^[^ ^115^, ^116^, ^117^, ^118^ ^]^ functional, 6–31G(d,p)^[^ ^119^, ^120^, ^121^, ^122^, ^123^, ^124^, ^125^ ^]^ basis set and the D3 version of Grimme's dispersion with Becke–Johnson damping (D3BJ).^[^ ^126^, ^127^ ^]^ This was then followed by a second optimization at the same level of theory in order to reorientate each molecule, as described below. Finally, the optimized and aligned geometry for each molecule was then taken as input for a time‐dependent DFT^[^ ^128^, ^129^, ^130^, ^131^, ^132^, ^133^, ^134^ ^]^ calculation using the Tamm–Dancoff approximation (TDA)^[^ ^135^ ^]^ solving for ten triplet and ten singlet excited states, at the same level of theory. The combination of the well‐established B3LYP functional,^[^ ^117^, ^118^, ^136^, ^137^ ^]^ in conjunction with the medium‐sized 6–31G(d,p) basis set^[^ ^119^, ^120^, ^121^, ^122^, ^123^, ^124^, ^138^, ^139^, ^140^, ^141^ ^]^ was chosen as a trade‐off between accuracy and computational cost; although more modern functionals and larger basis sets are available, the large number of calculations performed in this study (468) demanded a more efficient methodology to ensure completion of the study in a timely manner. The B3LYP functional has previously been used by many groups,^[^ ^24^, ^41^, ^46^, ^47^, ^49^, ^63^, ^80^, ^81^, ^93^, ^95^, ^111^, ^142^, ^143^, ^144^, ^145^, ^146^, ^147^, ^148^, ^149^, ^150^, ^151^, ^152^, ^153^, ^154^ ^]^ including our own, to both optimize geometries and calculate photophysical properties of organic emitters with acceptable accuracy and short wall times, and as such represented an ideal compromise. With this scheme, the average duration of each calculation was 2.72 h and the total duration for all computations was a relatively modest 1273.78 h. All calculations were performed using the Gaussian 16, Revision A.03 program in the gas phase and convergence criteria and integration grid size were left as default for that program.^[^ ^155^ ^]^ Analysis of the results was performed with the aid of the open‐source cclib library.^[^ ^156^ ^]^

The form factor of each molecule was then estimated from these calculations. Reorientation and alignment of the molecule to the Cartesian axes was performed in the first instance by the Gaussian 16 program according to symmetry rules. The coordinates of each molecule were then transformed through a series of 90° rotations around its center (as defined by Gaussian 16) so that the greatest maximum difference in coordinates was in the *x* axis, the second most in the *y*, and the least in the *z*. For each molecule, this yielded the following parameters:

- Dimension along the three principal axes, expressed as *x*, *y*, and *z*, respectively.
- Linearity, defined as *L* = 1 − (*y*/*x*).
- Planarity, defined as *P* = 1 − (*y*/*z*).

It is important to clarify that we henceforth refer to “linear” or “planar” molecules based only on *L* and *P* as defined above and as obtained from ground state DFT calculations. As we mentioned in the previous section, the literature frequently refers to linear or planar molecules based on their chemical structure (e.g., on‐axis D–A or D–A–D) without a clear quantification of linearity, planarity, or aspect ratio.

Other relevant parameters of the molecules, such as the amplitude of the permanent electric dipole moment (PDM) in the ground state were provided by DFT or TDDFT calculations while the TDM (S_1_→S_0_) were obtained through TDDFT calculations. Additionally, we extracted the orientation of each of these dipole moments with respect to the *x* axis of the molecules, as well as with respect to their *xy* plane. In the case of host–guest systems, we obtained the above parameters for both the host and the emitter molecules. Finally, we included other parameters that may influence molecular orientation, such as the doping concentration (by weight) of the emitter molecules (*N*_E_) and the MW of the host and the emitter (MW_H_ and MW_E_, respectively); as well as two parameters that depend on combined properties of host and guest molecules, namely *x*_E_/*x*_H_, the length ratio between the long axes of the emitter and the host, and *PDM*_HE_ = *PDM*_H_∙ρ_H_ + *PDM*_E_∙ρ_E_, a combined PDM of the film that takes into account the molar fractions of host and emitter molecules (ρ_H_ and ρ_E_).^[^ ^81^ ^]^

### 6.2 Statistical Analysis

In the absence of a physical or chemical model that predicts a particular functional relation between the parameters defined above and the anisotropy factor (*a*), we assumed a multiple linear model to identify parameters that have a meaningful influence on the orientation of the TDMs. This simplified model can be useful to identify independent parameters that significantly explain variations in other dependent parameters.^[^ ^157^, ^158^ ^]^ The first part of our analysis consisted of finding correlations between all input parameters and *a*. The strength of the correlations was assessed by the Pearson correlation coefficient (*r*) and by their *p*‐value (*p*).^[^ ^157^ ^]^ We then used all parameters *ξ_i_* that had statistically significant correlations (*p* < 0.05) in a multiple linear regression, expressing *a* as a linear function of these parameters:

(10) $$\begin{array}{c} a\;\left(\xi_{i}\right)=\underset{i}{\sum \;}b_{i}\;\xi_{i}+c \end{array}$$

From this step, we identified the most influential parameters based on the statistical significance of their coefficients (*b_i_*), iteratively discarding the variables with highest *p*‐values and running the regression again until all the *b_i_* satisfied *p_i_* < 0.05. Then, we sorted the remaining *ξ_i_* according to their standardized coefficients:

(11) $$\begin{array}{c} \beta_{i}=\frac{b_{i}\sigma_{\xi_{i}}}{\sigma_{a}} \end{array}$$

where $\sigma_{a}$ and $\sigma_{\xi_{i}}$ are the standard deviations in the values of a and ξ_*i*_, respectively. A larger value of β*_i_* represents a stronger influence of *ξ_i_* on a. Finally, we evaluated the quality of the multiple linear regressions based on their *F*‐test, *p*, and adjusted‐*R* ^2^ values (*F*, *p*, and ${\bar{R}}^{2}$, respectively).^[^ ^157^, ^158^ ^]^ The *F* from a regression with *k* independent variables (descriptors) and based on *n* observations (*F_k,n− k −_*_1_) is the ratio of the variance in the data that is explained by the linear model to the variance that is left unexplained. Thus, a larger *F* is related to a larger influence of the descriptors on the dependent variable. Similarly, ${\bar{R}}^{2}$ can be related to the fraction of the variation in *a* that can be explained by the variation in the proposed set of descriptors {*ξ_i_*}.^[^ ^158^ ^]^ The entirety of this analysis was done using the software Stata 14.^[^ ^159^ ^]^

## 7 Results from Meta‐Analysis of Literature Data and DFT Modeling

In an initial test, we fed the algorithm only with emitter‐related parameters from the systems included in this study (i.e., no host‐related data was considered). Clear correlations of *a* were observed with various parameters (*N*_E_, MW_E_, *L*_E_, and the dimensions of the emitter molecules, *x*_E_, *y*_E_, and *z*_E_). We note that the correlation between *a* and *N*_E_ is likely to come from clustering of the data, since we took *N*_E_ = 100 wt% for neat films and its largest value in doped films was 30 wt%. The only parameters with statistically significant coefficients in the multiple linear regression were MW_E_, *L*_E_, *z*_E_, and *N*_E_, in that order ($\beta_{{\text{MW}}_{E}}$ = −0.742, $\beta_{L_{E}}$ = −0.268, $\beta_{z_{E}}$ = 0.169, $\beta_{N_{E}}$ = −0.163). Importantly, while the regression was statistically significant (*F*_4198_ = 45.42, *p* < 0.00005), it accounted for only 47% of the variation in *a* (${\bar{R}}^{2}$ = 0.47). It can thus be expected that there are additional factors influencing the molecular orientation of the emitters (e.g., the *T*_g_ of the host molecules) and that the importance of these factors may depend on the nature of the deposited systems, that is, whether they are neat or doped. Thus, we analyzed the data from neat and doped films independently. To simplify the analysis of doped films, we only included data from systems that involved only one kind of host molecule (i.e., co‐hosts systems were not considered).

### 7.1 Neat Films

Neat films of purely organic emitter molecules are not frequently used as an EML in OLEDs due to aggregation‐caused quenching of the luminescence and the generally poorer charge‐transport properties of many emitters compared to host materials.^[^ ^160^ ^]^ This has limited the number of studies on the influence of molecular orientation on the efficiency of OLEDs based on non‐doped EMLs. However, over the past decade, there have been reports of blue and deep‐blue, fluorescence‐based OLEDs achieving beyond 6% EQE_max_ due to improved horizontal orientation of the emitters, together with efficiency enhancement via triplet–triplet annihilation and aggregation‐induced fluorescence.^[^ ^38^, ^56^, ^67^ ^]^ More recently, Shi et al. reported a device based on a neat film of the TADF material *m*TPy‐PXZ with an EQE_max_ of 23.6%, where the emitter adopted a preferentially horizontal orientation in the film (*a* = 0.24).^[^ ^68^ ^]^

In this section, we used the available data from 13 reports on the orientation of 26 emitter molecules in neat films to identify the most influential factors for TDM alignment in these systems. We left out the report by Hasegawa et al. on the orientation of 4CzIPN mentioned in Section 5 because, so far, the results obtained by depositing mono‐ and multilayers at ≈1 layer per 1000 s cannot be translated to practically relevant deposition rates (>0.1 Å s^−1^), rendering the growth of thicker films highly challenging.^[^ ^111^ ^]^

**Figure 10:**
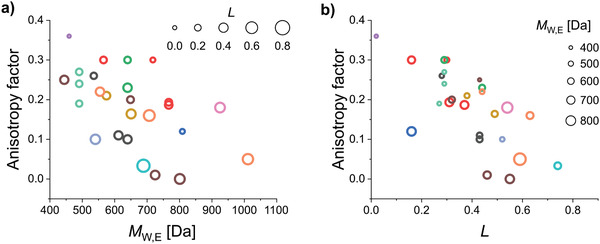
a,b) Anisotropy factor as a function of MW (a) and *L* (b) in neat film emitter systems. MW and *L* account for 54% of the variation in the anisotropy factor in this dataset. The color coding of the symbols corresponds to the groups of molecules shown in Figure 11. The size of the symbols in (a) and (b) is scaled as a linear function of *L* and MW, respectively. The circle sizes on the panel are a guide to the eye.

In the first step of our analysis, we found strong correlations of *a* with MW, *x*, and *L*. However, *x*, the long molecular axis, was strongly correlated with MW and *L* ($r_{x,\text{MW}}$ = 0.7851, *p* < 0.00005; *r*_*x*,*L*_ = 0.7736, *p* < 0.00005). Thus, the only parameters with statistically significant coefficients in the multiple linear regression were *L* and MW, in that order (β_*L*_ = −0.582, $\beta_{\text{MW}}$ = −0.312). (We also found a weak correlation between *L* and MW in the dataset [$r_{L,\text{MW}}$ = 0.3928, *p* = 0.0471].) **Figure** **10** shows plots of *a* as a function of MW and *L*, clearly illustrating the trends identified by our statistical analysis. The regression accounted for 54% of the variation in *a* (${\bar{R}}^{2}$ = 0.54) and was highly statistically significant (*F*_2,23_ = 15.73, *p* < 0.00005).

**Figure 11:**
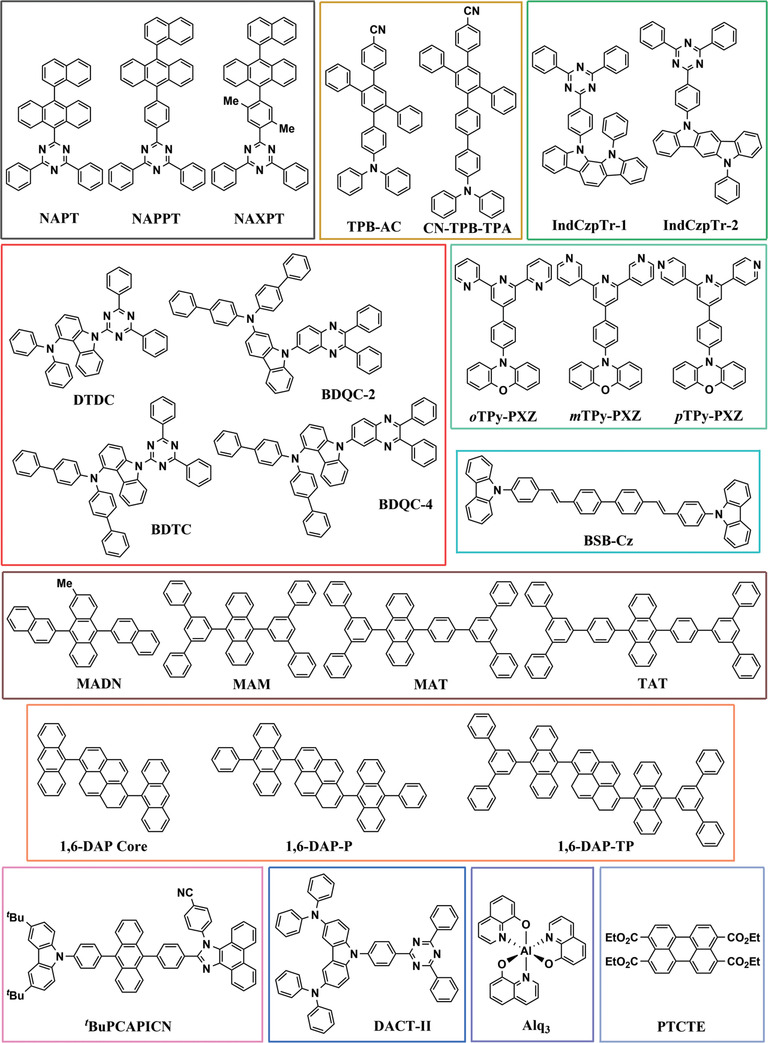
Molecular structure of the emitters included in this section.^[^ ^38^, ^41^, ^42^, ^52^, ^56^, ^61^, ^67^, ^68^, ^69^, ^74^, ^93^, ^110^, ^112^ ^]^ The color coding corresponds to the color of the symbols plotted in Figure 10.

The influence of *L* and MW on the orientation of emitters can be clearly seen in most of the individual studies that compared the orientation of at least two molecules with similar structures. For example, in a study by Kim et al.,^[^ ^38^ ^]^ the inclusion of a phenyl or xylene ring between the donor and acceptor units increased *L* and MW of NAPPT and NAXPT with respect to NAPT (dark gray circles in Figure 10, structures shown in **Figure** **11**). The higher MW of the xylene ring in NAXPT may account for the slightly improved orientation with respect to NAPPT. A similar case was recently reported by Han et al.,^[^ ^61^ ^]^ who improved the orientation of the molecule CN‐TPB‐TPA by the addition of a bridging phenyl ring to the reference molecule TPB‐AC (yellow circles in Figure 10, structures shown in Figure 11). (The parent structure TPB‐AC had been previously reported in a separate work by Xu et al.^[^ ^56^ ^]^) Similarly, in a different study by Kim et al.,^[^ ^41^ ^]^ the improved *L* and higher MW of BDQC‐2 and BDQC‐4 with respect to DTDC and BTDC result in a higher degree of horizontal orientation of their TDM (red circles in Figure 10, structures shown in Figure 11). It is worth noting that the higher MW of BTDC with respect to DTDC seems to be counter‐acted by its lower *L* and thus both molecules yielded similar degrees of horizontal orientation.

A case where the influence of *L* is seen more clearly is in a study by Xiang et al.,^[^ ^52^ ^]^ where the orientation of two indocarbazole isomers with different degrees of linearity is investigated (green circles in Figure 10, structures shown in Figure 11). Here, the higher *L* of IndCzpTr‐2 with respect to IndCzpTr‐1 noticeably improved its horizontal orientation. Interestingly, the most linear molecule of this dataset, BSB‐Cz (turquoise circle in Figure 10, structures shown in Figure 11),^[^ ^93^ ^]^ did not have the highest degree of horizontal orientation. Yokoyama et al. measured a value of *S* in neat films of BSB‐Cz equivalent to *a* = 0.03, which is already close to perfect horizontal orientation within the accuracy of the measurement. However, the highest degree of horizontal orientation in an emitter deposited as a neat film was reached by TAT, a molecule that combines a high *L* with a high MW.^[^ ^67^ ^]^ In the same study, the gradual increase in *L* and MW from MAM, through MAT, to TAT was found to led to a gradual increase in horizontal orientation (brown circles in Figure 10, structures shown in Figure 11).

A case where the influence of MW can be seen more clearly is that of the molecules 1,6‐DAP‐core, 1,6‐DAP‐P, and 1,6‐DAP‐TP, reported by Lee et al.^[^ ^112^ ^]^ The significantly different MW values of these molecules led to pronounced changes in their TDM orientation (orange circles in Figure 10, structures shown in Figure 11). It is worth noting that the orientation of 1,6‐DAP‐TP was more horizontal (*a* = 0.05) than that of 1,6‐DAP‐P (*a* = 0.16), even though the latter has a higher *L* (0.63) than the former (0.59).

Overall, according to the data currently available, MW and *L* seem to be the most influential parameters for horizontal orientation in neat films. The ${\bar{R}}^{2}$ of the regression improved from 0.54 to 0.60 by adding the normalized off‐*x* component of the TDM (*TDM*_off‐x_) of the S_1_→S_0_ transition as a regressor. Since linear emitters tend to orient more horizontally in neat films, the alignment of the TDM with the long axis of the molecules can be important for achieving horizontal TDM orientation. Indeed, the regression coefficient of this parameter was positive (β = 0.268), indicating a tendency of the TDM to lay more horizontally in the films when aligned closer to the long axis of the molecules (lower *a* for larger *TDM*_off‐x_). It should be noted that this coefficient had a lower statistical significance (*p* = 0.053) than the coefficients of *L* and MW in this regression (*p* = 0.001 and *p* = 0.013, respectively) and we did not observe a statistically significant correlation between *a* and *TDM*_off‐x_ (*r* = 0.1946, *p* = 0.3408). Thus, more data is required in order to assess the relative influence of *TDM*_off‐x_.

It is very likely that there are additional factors influencing orientation in neat films that were not incorporated into our model. An example of this is the difference in the orientation of the three isomers *o*TPy‐PXZ (*a* = 0.27), *m*TPy‐PXZ (*a* = 0.24), and *p*TPy‐PXZ (*a* = 0.19), reported by Shi et al. (light‐green circles in Figure 10, structures shown in Figure 11).^[^ ^68^ ^]^ The differences in orientation of these three molecules in neat films were attributed by the authors of that study to different packing arrangements arising from different hydrogen‐bonding interactions. These interactions have been previously shown to be a good strategy for guiding molecular alignment in organic materials^[^ ^32^ ^]^ and recent works by Shi et al.^[^ ^68^ ^]^ and Sasabe et al.^[^ ^63^ ^]^ have started to exploit them toward the horizontal orientation of TADF emitters. Unfortunately, we were not able to incorporate any parameter related to these interactions into our analysis due to the difficulty of quantifying the variables involved (packing structure, number of bonding sites, bonding strength, etc.) and due to the limited amount of related data.

### 7.2 Binary Host–Guest Systems

**Figure 12:**
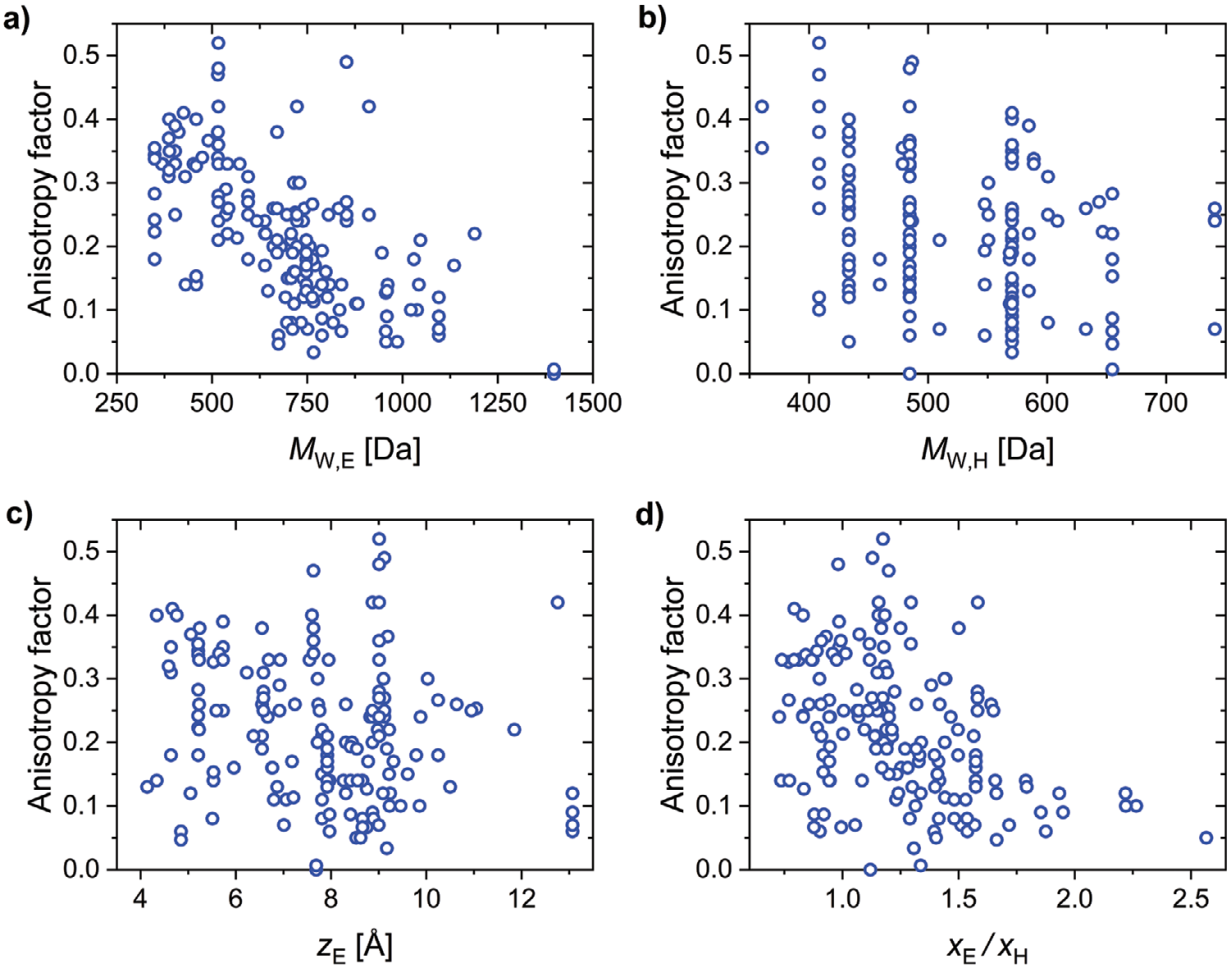
a–d) Factors that influence the TDM orientation of emitters in host–guest systems: a) MW_E_, b) MW_H_, c) *z*_E_, and d) *x*_E_/*x*_H_.

To identify the key parameters driving horizontal TDM orientation in the more widely used binary host–guest systems, we extended our set of parameters to cover hosts as well as emitters. This subset of data included 164 host–emitter systems. We found that the most significant parameters in the multiple linear regression for *a* were the MW_E_, MW_H_, *x*_E_/*x*_H_, and *z*_E_, in that order (${\bar{R}}^{2}$ = 0.55, *F*_4159_ = 51.74, *p* < 0.00005, $\beta_{{\text{MW}}_{E}}$ = −0.605, $\beta_{{\text{MW}}_{H}}$ = −0.364, $\beta_{x_{E}/x_{H}}$ = −0.309, $\beta_{z_{E}}$ = 0.216). The degree of horizontal TDM orientation in doped films increased with MW_E_ *_,_* MW_H_, and *x*_E_/*x*_H_ (**Figure** **12**). On the contrary, the higher the value of *z*_E_ (thicker molecules), the less horizontally oriented their TDM. We found statistically significant correlations of *x*_E_/*x*_H_ and *z*_E_ with MW_E_ ($r_{x_{E}/x_{H},\text{MW}}$ = 0.4232, *p* < 0.00005; $r_{z_{E},\text{MW}}$ = 0.6079, *p* < 0.00005) as larger emitter molecules inevitably have higher MW_E_. However, the regression coefficients of *x*_E_/*x*_H_ and *z*_E_ were statistically significant, and their inclusion in the regression led to a significant increase in ${\bar{R}}^{2}$ from 0.46 to 0.55, so we concluded that they are meaningful descriptors in addition to MW_E_.

Remarkably, every system that achieved *a* ≤ 0.10 (90% horizontal orientation) fulfilled at least one of the following two conditions: MW_E_ > 700 Da or *x*_E_/*x*_H_ > 1.3. Importantly, however, not every system that satisfied either of these conditions achieved *a* ≤ 0.1. To carefully explore the properties of the emitters that achieved *a* ≤ 0.10 and their relation to the strategies reviewed in Section 5, we have classified these molecules into three groups. Namely, i) molecules with high aspect ratios, ii) bulky molecules with high MW, and iii) molecules that achieved highly horizontal TDM orientation via favorable host–guest interactions. In the following section, we discuss these three strategies in the context of our meta‐analysis and DFT‐based quantification of molecular parameters.

#### 7.2.1 Molecules with High Aspect Ratios

**Figure 13:**
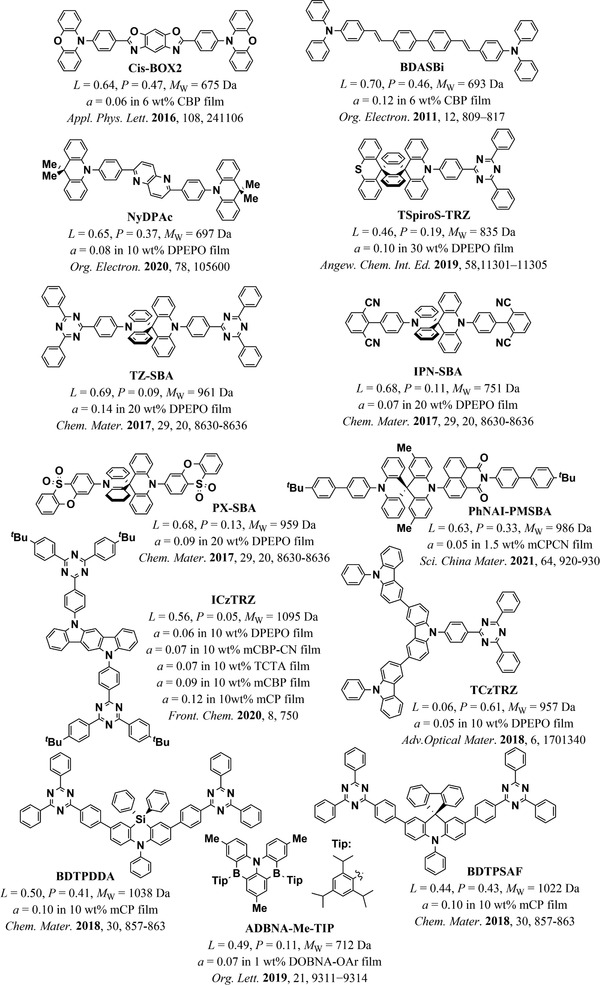
Emitters with large aspect ratios that achieved *a* ≤ 0.10 in host–guest systems. The molecular structure of BDASBi has been included here for comparison with *cis*‐BOX2. L and P refer to linearity and planarity as defined in the text.

As discussed in Section 4, increasing aspect ratio has been one of the main strategies to promote horizontal orientation of emitters in OLEDs (**Figure** **13**). A good example of this strategy is the molecule *cis*‐BOX2, which was shown by Komino et al. to achieve *a* = 0.06 when deposited at room temperature in a CBP host, pushing the EQE_max_ of OLEDs based on this system up to 27.8%.^[^ ^40^ ^]^ Remarkably, a perfect horizontal orientation within the precision of the measurement (*a* = 0) and an EQE_max_ of 33.4% was also shown by the authors when depositing the EML at low temperatures (200 K, not included in our meta‐analysis due to limited amount of data for deposition at non‐standard temperatures). *Cis*‐BOX2 has only a modest MW_E_ = 675 Da, but it has an *x*_E_ = 25.7 Å, one of the longest of all the molecules included in this section. When compared to the length of the CBP host, we get *x*_E_/*x*_H_ = 1.39. Additionally, *cis*‐BOX2 shows high linearity (*L* = 0.64) and planarity (*P* = 0.47), and the TDM is well aligned along the long molecular axis. All these factors are likely to play a role in the highly horizontal TDM orientation of *cis*‐BOX2 in CBP films. Interestingly, the longer molecule BDASBi (*x*_E_ = 30.6 Å, *L* = 0.70, *P* = 0.46) did not achieve a comparable horizontal orientation in CBP under otherwise identical conditions (*a*_BDASBi_ = 0.12, *a_cis_*_‐BOX2_ = 0.06).^[^ ^76^ ^]^ We propose that this may be due to the higher conformational rigidity of *cis*‐BOX2. More recently, Lee et al. demonstrated highly horizontal TDM orientation of the molecule NyDPAc doped in DPEPO (*a* = 0.08).^[^ ^64^ ^]^ NyDPAc has a comparable length and linearity to those of *cis*‐BOX2, but a lower planarity (*x*_E_ = 25.4 Å, *L* = 0.65, *P* = 0.37). Compared to DPEPO, it has *x*_E_/*x*_H_ = 1.54. Lee et al. used this host–emitter system in an OLED, which showed an EQE_max_ of 20.9%.

Over the past 3 years, various groups have reported other molecules that have pushed OLED efficiency beyond 30% without the need for deposition at low temperatures. For example, TspiroS‐TRZ has a lower aspect ratio than *cis*‐BOX2, but a higher MW (*x*_E_ = 21.7 Å, *L* = 0.46, *P* = 0.19, MW_E_ = 835 Da).^[^ ^48^ ^]^ Its subsequent highly twisted moieties, arranged in an elongated fashion, allowed Li et al. to achieve an EQE_max_ of 33.3% in sky‐blue TADF OLEDs based on TspiroS‐TRZ (*a* = 0.10, *x*_E_/*x*_H_ = 1.31 in DPEPO). Following a similar strategy, Liu et al. synthesized a family of four long molecules with high *L*.^[^ ^24^ ^]^ One of these (TZ‐SBA) was used in a device that reached 35.2% EQE_max_, albeit with a more modest horizontal orientation (*a* = 0.14). Two other molecules of this family, IPN‐SBA and PX‐SBA showed more horizontally oriented dipoles, with *a* = 0.07 and 0.09, respectively. However, the EQE_max_ of OLEDs based on IPN‐SBA and PX‐SBA was considerably lower (24.7% and 20.8%, respectively). All three molecules are highly linear (*L* = 0.69 for TZ‐SBA and *L* = 0.68 for IPN‐SBA and PX‐SBA), and have MW > 750 Da, and *x*_E_/*x*_H_ > 1.5 for the DPEPO host used in the study.

A similar molecule with a highly horizontal TDM orientation, the red emitter PhNAI‐PMSBA, was recently reported by Zeng et al.^[^ ^75^ ^]^ PhNAI‐PMSBA is the longest molecule in our database (*x*_E_ = 34.4 Å), it is highly linear (*L* = 0.63), and it also has a high MW (986 Da). Additionally, when used in the mCPCN host, PhNAI‐PMSBA achieved the highest *x*_E_/*x*_H_ value in this database (2.57). All of these properties are likely significant for achieving a TDM orientation of *a* = 0.05 in mCPCN. An OLED based on PhNAI‐PMSBA showed an EQE_max_ of 22.3%. Another molecule that combined a high aspect ratio with high MW was recently reported by Naqvi et al.^[^ ^82^, ^106^ ^]^ Remarkably, the sky‐blue TADF emitter ICzTRZ (*x*_E_ = 31.0 Å, *L* = 0.56, MW_E_ = 1095 Da) achieved *a* < 0.10 in four different hosts (mCBP, mCBP‐CN, DPEPO, and TCTA) and *a* = 0.12 in mCP. Importantly, it had *x*_E_/*x*_H_ > 1.56 with respect to all of them. It is worth noting that ICzTRZ had the longest *z*_E_ in our database (13.1 Å), which led to a low planarity value (*P* = 0.05). This was the only emitter with *z*_E_ > 9.5 Å in our database that achieved *a* < 0.05 in a binary host–guest system (cluster of points on the lower right of Figure 12c). ICzTRZ was used by Zhang et al. in an OLED that achieved an EQE_max_ = 22.1%.^[^ ^82^ ^]^ In a slightly different approach, seeking to increase the planarity rather than the linearity, Byeon et al. reported TCzTrz, a Y‐shaped molecule with high MW (957 Da) and relatively large size in *x* and *y* (*x*_E_ = 23.2 Å, *y*_E_ = 21.9 Å).^[^ ^47^ ^]^ Due to its Y‐shape, it has a very low linearity (*L* = 0.06) but the second‐highest planarity value of the molecules included in this study (*P =* 0.61). Byeon et al. found that TCzTrz reached *a* = 0.05 in DPEPO (*x*_E_ */x*_H_ = 1.40), which contributed to an EQE_max_ of 31.8% in the OLED based on this emitter.

Finally, other molecules that achieved *a* = 0.10 by following the large aspect ratio approach are BDTPDDA and BDTPSAF, two extended fluorescent molecules (*x* > 30 Å, *y* > 15 Å) with high MW (each ≈1 kDa) that were reported by Woo et al.^[^ ^105^ ^]^ Both have *L* and *P* values between 0.4 and 0.5. Although neither of these values are particularly high compared to the other molecules in this group, both molecules combine large *x*_E_ (> 30 Å) and high MW_E_, while retaining *L* and *P* values above 0.4. Additionally, both had *x*_E_/*x*_H_ > 2.2 when used in mCP. The high degree of horizontal TDM orientation they achieved in this host allowed to realize fluorescent OLEDs with an EQE_max_ value of 8.5%. Another system that achieved a highly horizontal emitter TDM was the multi‐resonant TADF emitter ADBNA‐Me‐Tip reported by Oda et al.^[^ ^73^ ^]^ It has a relatively low MW_E_ = 712 Da and *x*_E_/*x*_H_ = 1.06 (for the host DOBNA‐OAr; MW_H_ = 633 Da). The relatively high linearity of this emitter (*L* = 0.49) probably contributed to achieving a TDM orientation *a* = 0.07 in DOBNA‐OAr, leading to an EQE_max_ = 15.0% in the corresponding device.

#### 7.2.2 Bulky Disk‐Like Molecules with High Molecular Weight

**Figure 14:**
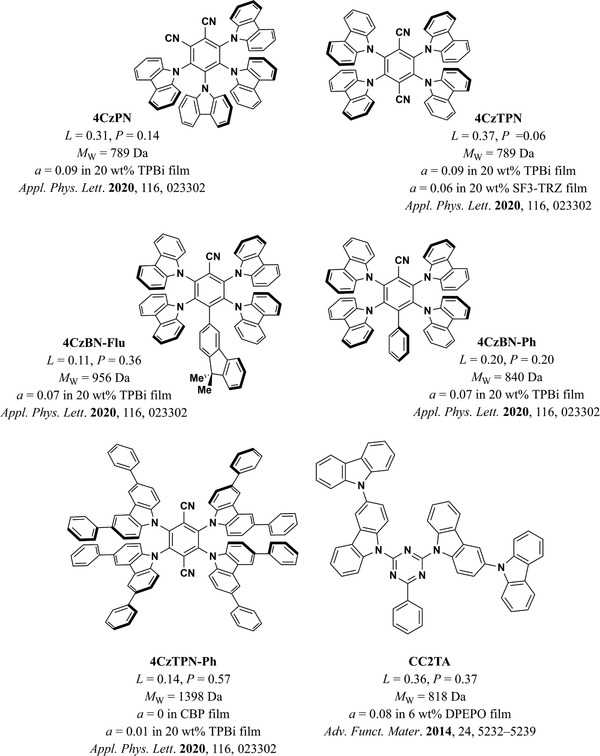
Molecular structure of bulky carbazole‐based emitters that achieved *a* ≤ 0.10 in host–guest systems.

An early report by Mayr et al. found that the bulky emitter CC2TA, which has a moderate aspect ratio, achieves a high degree of horizontal TDM orientation in DPEPO (*a* = 0.08, *x*_E_/*x*_H_ = 1.29) (**Figure** **14**).^[^ ^77^ ^]^ However, it was not until a recent study by Tanaka et al. that this strategy was explored more systematically.^[^ ^81^ ^]^ In this study, the authors looked at the orientation of a family of small, bulky, disk‐shaped TADF molecules with different permanent dipole moments (the effect of the permanent dipole moment will be discussed later in this review). All these molecules have particularly high MW. More specifically, all of those that achieved *a* < 0.10 had MW > 780 Da. Remarkably, 4CzBN‐Ph achieved *a* = 0.07 in TPBi despite having particularly low aspect ratios (*L* = *P* = 0.20, *x*_E_/*x*_H_ = 0.88), but a high MW = 840 Da. Similar orientation values were obtained by Tanaka et al. for other bulky molecules with low aspect ratios, such as 4CzTPN, 4CzPN, and 4CzBN‐Flu (see Figure 14), all of which had *x*_E_/*x*_H_ < 1 in the hosts in which they achieved *a* < 0.1. A further extension of the bulky substituents increased the MW and planarity of 4CzTPN‐Ph (MW = 1398 Da, *P* = 0.57), yielding a TDM orientation *a* = 0 in CBP (*x*_E_/*x*_H_ = 1.12) and *a* = 0.01 in TPBi (*x*_E_/*x*_H_ = 1.34). Although this strategy has not yet led to devices that achieve high levels of horizontal TDM alignment (*a* < 0.1) and high efficiency (EQE_max_ > 11%)^[^ ^77^ ^]^ at the same time, the high degree of horizontal orientation that these molecules achieve indicates that this may be a promising strategy.

#### 7.2.3 Favorable Geometries and Host–Guest Interactions of Emitters with Modest Molecular Weight and Aspect Ratios

**Figure 15:**
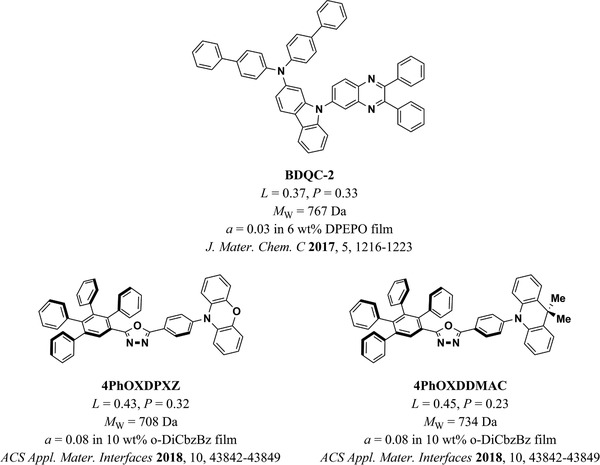
Molecular structures of emitters that achieved *a* ≤ 0.10 through favorable geometry‐based host–guest interactions.

This final group includes emitters that have shown low values of *a* without having high aspect ratios nor particularly high MW (**Figure** **15**). Instead, they have achieved high degrees of horizontal TDM orientation by means of combining beneficial host–guest interactions and complementary geometries. BDQC‐2 is part of a larger set of molecules from this category reported by Kim et al.^[^ ^41^ ^]^ It has only moderately high values of MW_E_ = 776 Da, *x*_E_ = 21.6 Å, *L* = 0.37, and *P* = 0.33. Nonetheless, it achieved almost perfectly horizontal TDM orientation when doped at 6 wt% into DPEPO (*a*_doped_ = 0.03, *x*_E_/*x*_H_ = 1.31). Importantly, the authors did not observe the same degree of orientation when depositing BDQC‐2 in neat films (*a*_neat_ = 0.19). Thus, it was concluded that the favorable orientation in DPEPO must be due to host–guest interactions. Lee et al. reported a similar case for the two emitters 4PhOXDDMAC and 4PhOXDPXZ, which have only modest values of MW_E_, *x*_E_, *L*, and *P* (MW_E_ =  734 Da, *x*_E_ = 21.0 Å, *L* = 0.45, *P* = 0.23 and MW_E_ = 708 Da, *x*_E_ = 20.0 Å, *L* = 0.43, *P* = 0.32, respectively).^[^ ^58^ ^]^ However, they achieved a highly horizontal TDM orientation in the host *o*‐DiCbzBz (*a* = 0.08, *x*_E_/*x*_H_ > 1.4 for both emitters). This was attributed to the complementary geometry of these molecules with the geometry of the host, inducing a favorable TDM orientation. Making use of this specific interaction, Lee et al. reported a device based on 4PhOXDPXZ with 29.2% EQE_max_.

We now add two further remarks regarding the findings from our dataset on binary host–guest systems. First, we repeated our analysis on a subset of our dataset, including only binary host–guest systems for which the *T*_g_ of the host is available (**Table** **1**). This subset contained 154 of the 164 binary systems in our dataset. As mentioned in Section 4, the importance of *T*_g_ has been previously explored in molecular orientation studies. We found a statistically significant correlation between *a* and *T*_g,H_ ($r_{a,T_{g,H}}$ = −0.1975, *p* = 0.0141). However, the correlation between *a* and MW_H_ was stronger ($r_{a,{\text{MW}}_{H}}$ = −0.3095, *p* = 0.0001). We also tested the significance of *T*_g,H_ and MW_H_ in different multiple linear regressions to this subset of data. The inclusion of either of these led to slightly different sets of statistically significant parameters, namely {MW_E_, *T*_g,H_, *x*_E_, *z*_E_} and {MW_E_, MW_H_, *x*_E_/*x*_H_, *z*_E_}. Both sets had very similar regression statistics and the related parameters had similar regression coefficients: ${\bar{R}}^{2}$ = 0.60, *F*_4149_ = 59.27, *p* < 0.00005, $\beta_{{\text{MW}}_{E}}$ = −0.747, $\beta_{T_{g,H}}$ = −0.309, $\beta_{x_{E}}$ = −0.231, $\beta_{z_{E}}$ = 0.271 for the former and ${\bar{R}}^{2}$ = 0.61, *F*_4149_ = 59.68, *p* < 0.00005, $\beta_{{\text{MW}}_{E}}$ = −0.692, $\beta_{{\text{MW}}_{H}}$ = −0.346, $\beta_{x_{E}/x_{H}}$ = −0.271, $\beta_{z_{E}}$ = 0.259 for the latter. The similar regression statistics for both sets are related to the fact that *T*_g_ is strongly influenced by MW and by the geometry of the molecules, as discussed previously.

**Table 1:** Host molecules in our database for which the *T*_g_ value is available in the literature

Host molecule	*T*_g_ [°C]	Reference
Alq3	175	^[^ ^36^ ^]^
BCP	62	^[^ ^36^ ^]^
BCPO	137	^[^ ^106^ ^]^
CBP	62	^[^ ^36^ ^]^
DPEPO	94	^[^ ^161^ ^]^
mCBP	92	^[^ ^106^, ^161^ ^]^
mCBP‐CN	113	^[^ ^106^ ^]^
mCP	65	^[^ ^106^ ^]^
mCPCN	97	^[^ ^162^ ^]^
*o*‐DiCbzBz	117	^[^ ^163^ ^]^
NPB	99	^[^ ^36^ ^]^
OXD‐7	77	^[^ ^36^ ^]^
PO_9_	122	^[^ ^106^ ^]^
PPT	107	^[^ ^161^ ^]^
SF3‐TRZ	135	^[^ ^81^ ^]^
Spiro‐2CBP	174	^[^ ^36^ ^]^
TCTA	151	^[^ ^36^ ^]^
TPBi	122	^[^ ^36^ ^]^

**Figure 16:**
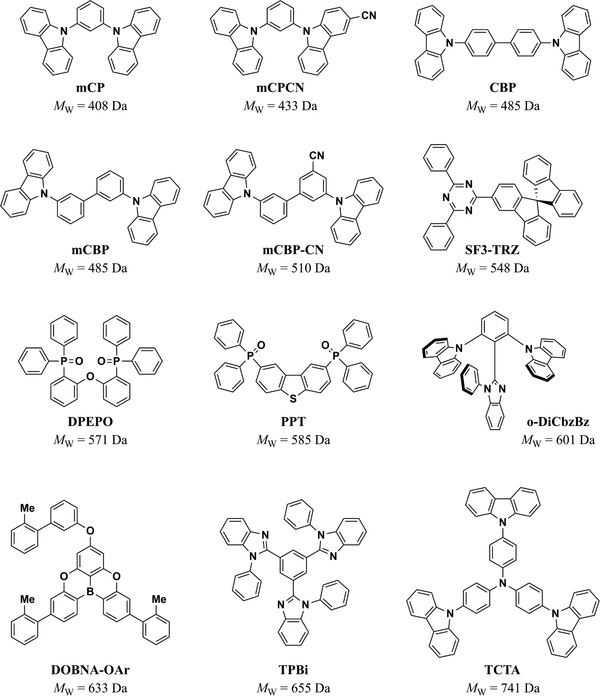
Molecular structure of the host molecules used in the binary host–guest systems that achieved an emitter orientation *a* ≤ 0.10.

Our second remark comes from three observations related to the influence of MW_E_ and MW_H_ on *a*. The only emitters that achieved *a* ≤ 0.10 in hosts with MW_H_ < 500 Da (CBP, mCBP, mCP, mCPCN; see **Figure** **16**) had either large aspect ratios (*cis*‐BOX2, BDTPDDA, BDTPSAF, ICzTRZ) or an exceptionally high MW_E_ (4CzTPN‐Ph, MW_E_ = 1398 Da); all emitters that achieved *a* ≤ 0.10 had MW_E_ > 600 Da; and the only emitters that adopted a TDM orientation of *a* > 0.20 in hosts with MW_H_ > 600 Da had MW_E_ < 600 Da. These three results indicate that dopant molecules with larger aspect ratios or high MW are less likely to diffuse and to be affected by the diffusion of the host molecules during film formation. This is in agreement with the recent report by Naqvi et al., who observed that the TDM orientation of the smaller TADF molecule DMAC‐TRZ was more dependent on the properties of the host than the larger emitter ICzTRZ.^[^ ^106^ ^]^ Due to the spread of MW_E_ values in our dataset, it is possible to define two separate categories in order to further study the factors that may influence the TDM orientation of emitters in host–guest systems: those with MW_E_ < 600 Da (low‐MW emitters) and those with MW_E_ > 600 Da (high‐MW emitters).

### 7.3 Low‐Molecular‐Weight Emitters

**Figure 17:**
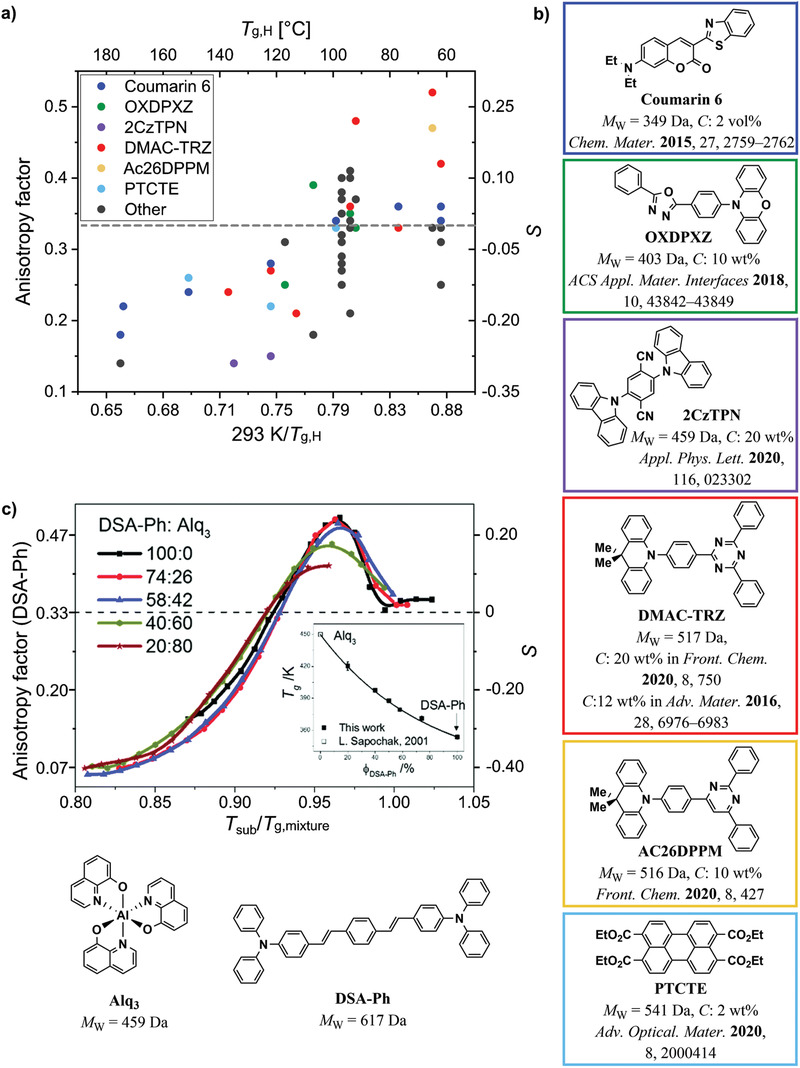
a) Anisotropy factor in host–guest systems as a function of 293 K per *T*_g,H_ for emitter molecules with MW < 600 Da. Emitters that were studied in three or more hosts are highlighted by colored symbols. The scale on the top shows the corresponding values of *T*_g,H_. The scale on the right shows the values of the order parameter (*S*) corresponding to the values of *a* shown on the left. The gray line corresponds to isotropic orientation. b) Structure of the molecules highlighted in (a). The MW of each molecule and the doping concentration (*C*) used in each study are shown for reference. c) TDM orientation of the molecule DSA‐Ph in a binary mixture with Alq_3_ as a function of *T*_substrate_/*T*_g,mixture_.^[^ ^107^ ^]^ The shape of the curve resembles the trend in (a). The scale on the right shows the values of S measured by Jiang et al. The scale on the left shows the equivalent values of *a*. The graph in (c) is adapted with permission.^[^ ^107^ ^]^ Copyright 2016, The Royal Society of Chemistry.

As mentioned above, low‐MW emitters are more susceptible to the diffusion of the host during film formation. This is consistent with the results of our analysis; we found that the only parameter with a statistically significant coefficient in the multiple regression for this subset of orientation data was the *T*_g_ of the host (${\bar{R}}^{2}$ = 0.40, *F*_1,55_ = 37.86, *p* < 0.00005), as shown in **Figure** **17**a. The subset of data used for the regression consisted of 57 host–emitter systems. We note that we have excluded the data of the orientation of ADBNA‐Me‐Mes in DOBNA‐OAr from this dataset due to the absence of data relating to the *T*_g_ of the host.^[^ ^73^ ^]^ This was the only case for a system with MW_E_ < 600 Da.

The *T*_g_ of the host alone accounted for 40% of the variation in the data. It is important to note that this result is largely influenced by two systematic studies by Brütting et al. in which they measured the TDM orientation of coumarin 6^[^ ^36^ ^]^ and DMAC‐TRZ^[^ ^106^ ^]^ in hosts spanning a wide range of *T*_g_ values. In both studies, the authors found that the TDM of the emitters aligns more horizontally when the molecules are doped in hosts with higher *T*_g_. This is largely in agreement with the extensive work of Ediger et al., who studied the molecular orientation of organic molecules deposited at different temperatures and found that this is mainly governed by the ratio of the temperature of the substrate to the *T*_g_ of the film (*T*_substrate_/*T*_g_).^[^ ^35^, ^164^ ^]^ For example, Jiang et al. studied the dependence of the TDM orientation of the molecule DSA‐Ph co‐deposited in binary films with Alq_3_ (Figure 17c).^[^ ^107^ ^]^ The TDM orientation of DSA‐Ph was more horizontal (low values of *a*, equivalent to low values of *S* according to Equations (7) and (9) for lower values of *T*_substrate_/*T*_g,mixture_; *a* gradually increased with this ratio until it reached isotropic orientation at *T*_substrate_/*T*_g,mixture_ ≈ 0.92, then reached a maximum (preferentially vertical orientation) at *T*_substrate_/*T*_g,mixture_ ≈ 0.96, before finally decreasing again toward isotropic orientation for higher values of *T*_substrate_/*T*_g,mixture_. Importantly, Jiang et al. observed that this trend in the TDM orientation of DSA‐Ph was independent of the concentration of the molecule in the film over a range from 20 to 100 wt%, even though the exact crossing point from preferentially horizontal to preferentially vertical orientation and the height of the maximum in *a* depended on the film composition.

The datapoints in Figure 17a follow the trend of the order parameter as a function of *T*_g_/*T*_substrate_ observed by Jiang et al. For a clearer comparison, we have added similar scales to the top and right of Figure 17a, assuming *T*_substrate_ = 293 K (room temperature) and a *T*_g_ of the mixture similar to that of the host. We can see that there is a discrepancy in the crossing point of the data through the isotropic orientation line between this dataset and the study by Jiang et al. This discrepancy may be partially due to the assumption of *T*_substrate_ = 293 K, but also due to the dependence of *T*_g,mixture_ on the doping concentration (inset in Figure 17c) and on the properties of the emitter (MW and geometry),^[^ ^113^, ^114^ ^]^ as well as on potential host–emitter and emitter–emitter interactions.^[^ ^35^ ^]^

While the *T*_g_ of the host was the only parameter with a statistically significant coefficient in our multiple regression of the low‐MW_E_ dataset, it only accounted for 40% of the variation in *a*. This is partly due to the fact that *a* does not follow a linear relation with respect to *T*_g_.^[^ ^107^, ^108^, ^109^ ^]^ However, it is still very likely that either there are additional parameters at play or that the size of the dataset is not sufficiently large to yield statistically significant conclusions. Apart from the influence of MW_E_ described above, another parameter that may be relevant is *x*_E_/*x*_H_. An indication of this can be seen from the systematic study by Mayr et al., which includes the orientation of coumarin 6 doped in two hosts with very similar *T*_g_, namely Alq_3_ (175 °C) and Spiro‐2CBP (174 °C).^[^ ^36^ ^]^ For these two hosts, the authors measured significantly different *a* values for coumarin 6; 0.18 for Alq_3_ and 0.22 for Spiro‐2CBP. This difference could be accounted for by the difference in *x*_E_/*x*_H_ between the two guest–host combinations; 0.89 for Spiro‐2CBP and 1.46 for Alq_3_. As discussed above, *x*_E_/*x*_H_ was a significant parameter for the orientation of emitters in the full set of binary host–guest systems. Further studies are required to clarify the influence of *x*_E_/*x*_H_ on the orientation of emitters doped in hosts with similar *T*_g_. Another possible reason for the difference in orientation of coumarin 6 in Alq_3_ and Spiro‐2CBP can be explained by electrostatic host–guest interactions, as recently pointed out by Bagchi and Ediger.^[^ ^35^ ^]^ According to our DFT calculations, coumarin 6 and Alq_3_ have large PDMs (4.8 and 8.1 D, respectively). By contrast, Spiro‐2CBP has almost no PDM (<0.1 D).

### 7.4 High‐Molecular‐Weight Emitters

We found that the most significant parameters in a multiple linear regression for *a* on the high‐MW_E_ dataset (a total of 106 host–emitter systems) were MW_E_, MW_H_, *x*_E_/*x*_H_, and *z*_E_, that is, the same as for the full dataset of binary systems, albeit with different regression coefficients (${\bar{R}}^{2}$ = 0.35, *F_4101_* = 14.85, *p* < 0.00005, $\beta_{{\text{MW}}_{E}}$ = −0.309, $\beta_{{\text{MW}}_{H}}$ = −0.444, $\beta_{x_{E}/x_{H}}$ = −0.359, $\beta_{z_{E}}$ = 0.234). As was the case in the complete dataset of binary host–guest systems, high MW of both emitters and hosts and high values of *x*_E_/*x*_H_ led to horizontally oriented TDMs of the emitter, while thinner emitter molecules (lower *z*_E_) also tended to orient with their TDM lying more horizontally.

**Figure 18:**
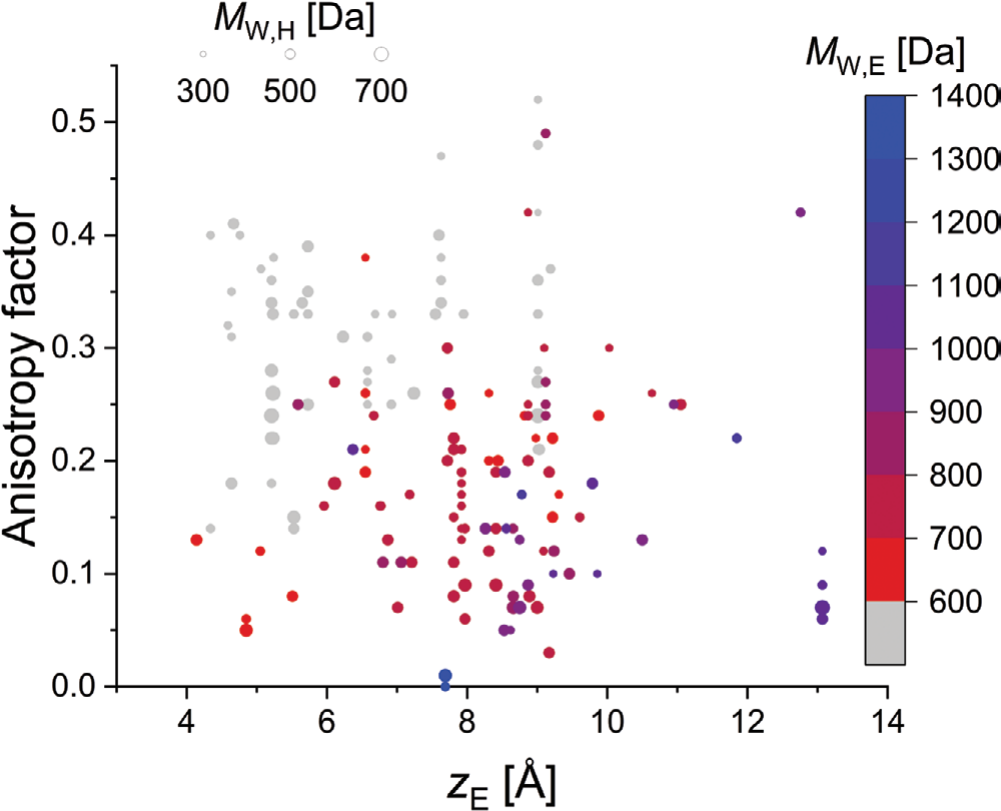
Anisotropy factor as a function of *z*_E_ (thickness of the emitters). The color coding indicates the molecular weight of the emitter and the symbol size indicates the molecular weight of the host. The data of low‐MW emitters in doped films has been added in light gray for reference. The cluster of datapoints on the lower right corner at *z*_E_ = 13.1 Å corresponds to the emitter ICzTRZ doped in different hosts.^[^ ^106^ ^]^

We note here that we neither found *L* nor *P* to be good descriptors for the variation in *a* in this subset of our data, nor in any of the other subsets related to host–guest systems. *x*_E_/*x*_H_ is strongly correlated to *L*. However, the former seems to be a better descriptor because it includes the interplay between the relative size of host and emitter molecules. In a similar way, the influence of *z*_E_ on *a* agrees with the commonly accepted view that more planar emitters tend to align more horizontally in the film (Figure 4a). However, the parameter that we believe defines planarity best (*P *= 1 − (*y*/*z*)) did not have any statistically significant correlation with *a* in any of our datasets. It is possible that *z*_E_ is a good descriptor due to its simultaneous correlation to MW_E_ and anticorrelation to both *P* and *L*. In any case, its influence on *a* became more apparent for the subset of high‐MW emitters (**Figure** **18**).

We repeated this analysis using data from systems with MW_E_ > 600 Da for which the *T*_g_ of the host is available (a total of 97 systems). We tested the significance of this parameter in the regression and compared it to that of MW_H_ in this subset of data. The inclusion of either *T*_g,H_ or MW_H_ led to the following two possible sets of significant parameters {MW_E_, *T*_g,H_, *x*_E_, *z*_E_} and {MW_E_, MW_H_, *x*_E_/*x*_H_, *z*_E_}. These are the same sets that we found when we used the full set of binary host–guest systems (see above). However, in this case we found that using *T*_g,H_ led to a noticeably lower ${\bar{R}}^{2}$ than using MW_H_: ${\bar{R}}^{2}$ = 0.36, *F*_4,92_ = 14.68, *p* < 0.00005, $\beta_{MW_{E}}$ = −0.459, $\beta_{T_{g,H}}$ = −0.333, $\beta_{x_{E}}$ = −0.277, $\beta_{z_{E}}$ = 0.362 for the former, and ${\bar{R}}^{2}$ = 0.42, *F*_4,92_ = 18.15, *p* < 0.00005, $\beta_{{\text{MW}}_{E}}$ = −0.429, $\beta_{{\text{MW}}_{H}}$ = −0.441, $\beta_{x_{E}/x_{H}}$ = −0.319, $\beta_{z_{E}}$ = 0.308 for the latter. Thus, MW_H_ was a better descriptor than *T*_g,H_ for this subset of our data.

As mentioned previously, during the process of film formation, molecules that land on the substrate or on the bulk of the film can undergo significant surface diffusion, even if the bulk of the film is below its *T*_g_.^[^ ^28^, ^165^ ^]^ The *T*_g_ of the film can still be a good reference point for the rate of this process. However, in host–guest systems this process depends on the properties of both the host and the emitter molecules, as well as on the doping ratio.^[^ ^81^, ^107^, ^108^, ^109^ ^]^ Low‐MW emitters doped at low concentrations in host matrices are less likely to have an impact on this process, as was observed by Mayr et al. for coumarin 6 doped at 2 wt% into different host matrices.^[^ ^36^ ^]^ Nonetheless, heavy guest molecules can significantly slow down the surface diffusion, especially if they are used at high concentrations. In this case, the *T*_g_ of the film can be very different to the *T*_g_ of the host.^[^ ^81^ ^]^ Therefore, the *T*_g_ of the film might be a better predictor for the orientation of the emitters than the *T*_g_ of the host alone, as was shown by Jiang et al.^[^ ^107^ ^]^ This may be the reason why MW_H_ and MW_E_ were better predictors in our multiple regression analysis than the *T*_g_ of the host. Unfortunately, the *T*_g_ of the host–guest system is rarely reported in studies focusing on the orientation of emitters and this conjecture therefore requires future experimental verification. Nonetheless, our analysis shows that MW_E_ is a key—frequently overlooked—parameter for achieving horizontal orientation of emitters, and that it is important to consider the interplay between the properties of the emitter and those of the host in emitter TDM orientation studies.

### 7.5 New Directions from Electrostatic Interactions

**Figure 19:**
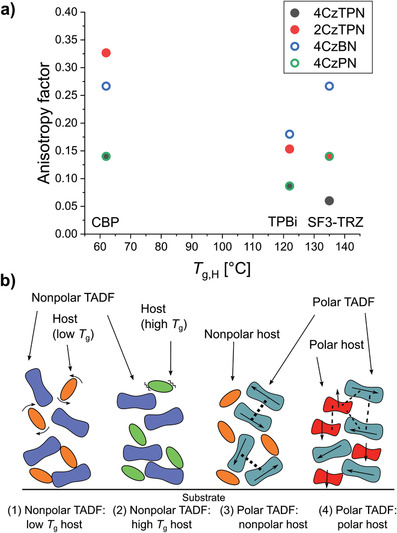
Influence of the polarity of host and emitter molecules. a) Anisotropy factor as a function of the host *T*_g_ for two non‐polar emitters (4CzTPN and 2CzTPN) and for two polar emitters (4CzBN and 4CzPN) in hosts with different *T*_g_. CBP and SF3‐TRZ are mostly non‐polar, whereas, by comparison, TPBi has a higher polarity. The figure was produced with data from ref. ^[^ ^81^ ^]^. b) Illustration of different interactions between polar and non‐polar host and guest molecules in TADF host–guest systems, as proposed by Tanaka et al. b) Adapted with permission.^[^ ^81^ ^]^ Copyright 2020, The Authors, published by AIP Publishing.

In the last section of this review, we turn our attention to recent studies on the influence of electrostatic intermolecular interactions on the orientation of emitters in OLEDs, as well as to what we can learn about them from our analysis. The influence of the PDM on the orientation of organometallic emitters has previously been a subject of intense discussion.^[^ ^95^, ^98^, ^166^, ^167^ ^]^ Purely organic fluorescent and TADF emitters are often of low symmetry and composed of donor and acceptor moieties that can lead to significant PDMs. However, this factor has not received much attention thus far in studies regarding the molecular orientation of purely organic emitters. In a recent report, Tanaka et al. studied the orientation of bulky, disk‐shaped TADF molecules with different polarities dispersed in CBP (*T*_g_ = 62 °C, *PDM* = 0.1 D), TPBi (*T*_g_ = 122 °C, *PDM* = 1.9 D), and SF3‐TRZ (*T*_g_ = 135 °C, *PDM* = 0.4 D).^[^ ^81^ ^]^ These hosts span a wide range of *T*_g_ and have different polarities (**Figure** **19**a). The authors found that the apolar emitters 4CzTPN (*PDM* = 0.0 D) and 2CzTPN (*PDM* = 0.0 D) followed a similar trend as that observed by Mayr et al. for coumarin 6,^[^ ^36^ ^]^ that is, an improved horizontal orientation of the emitters in hosts with higher *T*_g_. By contrast, the polar emitters 4CzPN (*PDM* = 7.2 D) and 4CzBN (*PDM* = 3.5 D) had the same degree of horizontal orientation in CBP and SF3‐TRZ, and a more horizontal orientation in TPBi. Thus, the authors proposed that dipolar interactions between TPBi (polar host) and polar guest molecules influence the orientation of these emitters by disrupting interactions between emitter molecules that would otherwise pair with anti‐parallel PDMs (Figure 19b). Strong emitter–emitter interactions have been previously suggested to be detrimental to the orientation of molecules that would otherwise orient horizontally due to weak host–guest interactions.^[^ ^93^ ^]^ Conversely, their study showed that dipolar host–emitter interactions can promote horizontal molecular orientation of emitters.

As mentioned in Section 6, we included PDM‐related parameters into our analysis for both hosts and emitters, including the magnitude of the PDM, its angle with respect to the *x* axis and with respect to the *xy* plane, as well as its off‐*x* and off‐*xy* components. We did not find any of these parameters to be significant in the regression for neat films. However, we found that the regression for the full subset of binary host–guest systems could be slightly improved by the addition of the off‐*xy* component of the PDM of the host (*PDM*_z,H_), yielding ${\bar{R}}^{2}$ = 0.58, compared to ${\bar{R}}^{2}$ = 0.55 without its addition. The regression coefficient of *PDM*_z,H_ was negative, −0.169, which meant that there was a tendency for the TDM of emitters to align more horizontally in systems with higher *PDM*_z,H_. It is possible that this improvement in the regression is due to the strong correlation between *T*_g,H_ and *PDM*_z,H_ (*r* = 0.5472, *p* < 0.00005), as well as to the paucity of data from studies involving hosts with *PDM*_z,H_ > 1.5 D (Alq_3_, mCBP‐CN, BCPO, TPBi). These constituted only 9% of the host–guest systems in our dataset.

The influence of *PDM*_z,H_ became even stronger in systems involving low‐MW emitters (<600 Da). In this case, replacing *T*_g,H_ by MW_H_ and *PDM*_z,H_ as regressors led to a modest improvement in ${\bar{R}}^{2}$ from 0.40 to 0.43. Both MW_H_ and *PDM*_z,H_ had strong correlations with *T*_g,H_, but no correlation between them. We speculate that the slight improvement in the regression may be related to the possibility of separately accounting for host–emitter interactions as well as for an increase in *T*_g,H_ by splitting this parameter into MW_H_ and *PDM*_z,H_. The regression could be improved even further to ${\bar{R}}^{2}$ = 0.46 by addition of the off‐*x* component of the emitter PDM as a regressor (in this case, the TDM orientation was less horizontal for emitters with larger off‐*x* component of their PDM). However, the significance of the regression coefficient of this parameter was low (*p* = 0.054).

In the case of the subset of data related to high‐MW emitters, including the magnitude of the PDM of the emitters as a regressor improved ${\bar{R}}^{2}$ from 0.35 to 0.37. This parameter had a statistically significant, negative regression coefficient. Thus, emitters with stronger PDM tended to orient with their TDM laying more horizontally in the film, even though this effect was shadowed by the other factors discussed above (MW_E_, MW_H_, *x*_E_/*x*_H_, *z*_E_). In this subset of our data, none of the parameters related to the PDM of the host was found to be significant. Due to the limited amount of available data, it is not possible to elucidate whether these improvements to the description of the variation of *a* in our dataset come from interactions related to the PDM of the emitter and host molecules. Thus, more studies are required that are specifically designed to probe these interactions.

In another recent publication, Naqvi et al. studied the orientation of DMAC‐TRZ in hosts with different *T*_g_ and polarity.^[^ ^106^ ^]^ They measured the degree of orientational order in the PDM of the host (*Λ*) and found that the TDM orientation of DMAC‐TRZ is strongly correlated to this parameter. The authors related this correlation to the degree of alignment of the host material and concluded that the latter can be an additional parameter for promoting the orientation of TADF emitters. Therefore, we built another subset of our data containing only the 73 host–emitter systems involving the hosts BCP, mCP, OXD‐7, mCBP, DPEPO, mCBP‐CN, BCPO, PO_9_, and TCTA, for which Naqvi et al. measured *Λ*. The regression was only slightly improved by adding *Λ* to the descriptors set {MW_E_, MW_H_, *x*_E_/*x*_H_, *z*_E_}, from ${\bar{R}}^{2}$ = 0.63 to ${\bar{R}}^{2}$ = 0.64, and there was no improvement at all when considering the subset with high‐MW emitters only. However, including *Λ* in addition to *T*_g.H_ as regressors to the data with low‐MW emitters had a more significant improvement in the regression, from ${\bar{R}}^{2}$ = 0.42 to ${\bar{R}}^{2}$ = 0.46. We note that using MW_H_ and *PDM*_z,H_ instead of *T*_g,H_ and *Λ* led to a higher ${\bar{R}}^{2}$ = 0.48 in the same dataset. *Λ* was correlated to MW_H_, *T*_g,H_, and *PDM*_z,H_ in all of these subsets of our data. Thus, its effect is hard to disentangle from those of the other variables. By contrast, using the combination of descriptors MW_H_ and *PDM*_z,H_ has the advantage that they are not correlated in any dataset. Unfortunately, given the paucity of studies on the orientation of a single emitter in multiple hosts, it is not possible to draw robust conclusions from these results.

**Figure 20:**
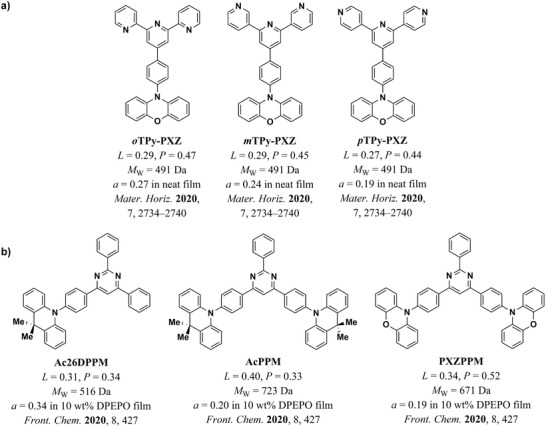
TADF emitters that showed improved orientation by making use of hydrogen‐bonding interactions. a) Isomers reported by Shi et al.^[^ ^68^ ^]^ b) Pyrimidine‐based TADF emitters reported by Sasabe et al.^[^ ^63^ ^]^

Finally, another kind of intermolecular interaction that can be relevant for the orientation of emitters in OLEDs is the formation of hydrogen bonds between neighboring molecules. This kind of interactions has been extensively studied in hosts and charge‐transport materials by Kido et al.,^[^ ^32^ ^]^ and has recently received increasing attention for improving the orientation of emitters. For example, Shi et al. studied the orientation of the three isomers *o*TPy‐PXZ, *m*TPy‐PXZ, and *p*TPy‐PXZ in neat films (**Figure** **20**a).^[^ ^68^ ^]^ They found that the modulation of the hydrogen‐bonding interactions can lead to improved horizontal TDM orientation. In a separate recent report, Sasabe et al. studied the orientation of the three emitters Ac26DPPM, AcPPM, and PXZPPM in doped films using mCP, CBP, mCPCN, and DPEPO as hosts (Figure 20b).^[^ ^63^ ^]^ They found that all of these emitters had a higher degree of horizontal TDM orientation in the host DPEPO, and that PXZPPM achieved the highest degree of TDM alignment. The authors concluded that this was due to the strong hydrogen‐bonding interactions between the strong electron‐withdrawing groups P=O of DPEPO and the CH groups in the emitters. They also associated the low degrees of horizontal TDM orientation of the emitters in mCP to the shorter π‐conjugation and lack of groups capable of making hydrogen bonds with the emitters. While we did not include any parameter accounting for the hydrogen‐bonding interactions in our analysis, we believe that further research in this direction will be useful for determining a full set of parameters that can describe emitter orientation in OLEDs.

## 8 Conclusions and Perspective

Over the last decade, intense research on the factors that influence horizontal alignment of the emitter TDM in OLEDs has led to the identification of guidelines that currently influence the design of organic fluorescent and TADF emitters. However, the extent to which these guidelines influence the emitter orientation, as well as a complete description of this process remain open questions to date. The significant increase in the number of reports over the past years has now allowed for a cross‐comparison of their results. In this review, we highlighted and discussed the main strategies for achieving highly oriented emitters in OLEDs that have been followed so far. Additionally, we presented the results of a statistical meta‐analysis of the orientation of fully organic emitters deposited in vacuum that is based on data from 203 different emitter systems.

We found that the MW and the linearity of the molecules are good descriptors for the orientation of the emitter TDM in neat films. A linear combination of these two uncorrelated parameters accounted for 54% of the variation of the anisotropy factor in this dataset. Indeed, the only reported emitter molecule that achieved 100% horizontal orientation in a neat emitter film in a working OLED (TAT) combined a high linearity and a high MW.^[^ ^67^ ^]^ By contrast, we found that the most influential factors for the orientation of emitters in binary host–guest systems are the MW of emitter and host, the thickness of the emitter, and the ratio between the lengths of the emitter and the host molecules. According to the results of our analysis, heavier and thinner emitters (small *z*_E_) tend to achieve higher degrees of horizontal orientation of their TDMs. Similarly, emitter molecules that are longer than the host molecules seem to be less affected by orientation‐scrambling diffusion processes during film deposition and so tend to be more horizontally oriented. Finally, we also found that hosts with higher MW tend to promote horizontal alignment of the emitter.

Different guidelines may apply for host–guest systems depending on the MW of the emitter. For light emitter molecules (MW < 600 Da), the most influential factor is the *T*_g_ of the host. This parameter alone accounted for 40% of the variation in *a* in this subset of our data. For heavy molecules (MW > 600 Da), the most influential parameters are the MW of the emitters, their molecular thickness, the MW of the host molecules, and the ratio between the length of the emitter and the length of the host. Higher degrees of horizontal orientation are achieved with longer, heavier, and thinner emitter molecules doped into heavier hosts.

The set of parameters identified by the multiple‐linear‐regression analysis accounted for 54% of the variation of the anisotropy factor in neat film systems, and for 55% of the variation in host–guest systems. Thus, additional research is required to elucidate further potential parameters that drive horizontal orientation of organic emitters in OLEDs. In particular, the field would benefit from measurements of the *T*_g_ of the host–guest systems (even though it will be technically challenging to obtain mixtures with a thermal history comparable to a thin vacuum‐deposited film) and from further studies on the influence of host–guest and guest–guest interactions in doped films. For now, we expect our results to provide a basis for future research that can, first, identify the full set of parameters that drive the horizontal orientation of organic emitters and, second, provide more cohesive guidelines for emitter design that can boost the outcoupling efficiency to produce highly efficient OLEDs.

## Conflict of Interest

The authors declare no conflict of interest.

## Data Availability

The data that support the findings of this study are openly available in the University of St Andrews research portal at https://doi.org/10.17630/ae55e842‐6db8‐4d3b‐975c‐b1905a0a45f3, ref. [168].
